# Enhancing covert communication in NOMA systems with joint security and covert design

**DOI:** 10.1371/journal.pone.0317289

**Published:** 2025-01-13

**Authors:** Thanh Binh Doan, Tien-Hoa Nguyen

**Affiliations:** 1 Department of Electronics and Telecommunications, Electric Power University, Hanoi, Vietnam; 2 School of Electrical and Electronic Engineering, Hanoi University of Science and Technology, Hanoi, Vietnam; Ozyegin University: Ozyegin Universitesi, TÜRKIYE

## Abstract

The explosion of Internet-of-Thing enables several interconnected devices but also gives rise chance for unauthorized parties to compromise sensitive information through wireless communication systems. Covert communication therefore has emerged as a potential candidate for ensuring data privacy in conjunction with physical layer transmission to render two lines of defense. In this paper, we aim to enhance the individual transmission of nearby users in non-orthogonal multiple access (NOMA) systems under scenarios of an eavesdropper who monitors covert transmission before decoding covert information. For this problem, we first provide a comprehensive analysis of the NOMA system in terms of outage probability (OP), secrecy outage probability (SOP), and detection error probability (DEP), where all of them are quantified in exact and asymptotic closed-form expressions. Besides, we have also derived closed-form formulas for users’ covert and public rates. Under the system requirements of the maximal OP and SOP and the minimal DEP, we formulate the optimization of resource power allocation to: 1) minimize the OP of covert communication and 2) maximize the covert rate. Thanks to the developed analytical expressions, we obtain closed-form expressions for the sub-optimal power allocation coefficient for each problem. Simulation results validate the efficacy of the analytical mathematical frameworks and reveal that the proposed approaches of power allocation can provide attractive performance improvement compared to fixed power allocations only.

## 1 Introduction

### 1.1 Background and motivation

Internet-of-Things (IoT), a new paradigm shift in wireless communication, has recently opened a new chapter for linking billions of devices across domains, where the widespread adoption of IoT devices yields several remarkable improvements in efficiency, automation, and data-driven decision-making. However, this raises critical concerns of security [1], covert communications [2], and reliability [3]. The main reason for these trends mainly stems from the design limitations of IoT devices in terms of computing power, processing power, and resilience, which makes their data transmission vulnerable to malicious attacks, such as jamming, poisoning, and denial-of-service (DoS) [1]. Especially, due to the simple encryption of communication data, any information transmitted by IoT nodes can be easily captured by eavesdroppers capable of strong processing capability through eavesdropping and monitoring actions. Even if traditional encryption methods can be used to challenge the eavesdropper’s decryption, sensitive information can still be revealed by analyzing metadata components, and a prime example of this is network traffic patterns [4]. Another example is in defence scenarios, where communications need to be both secure and covert, ensuring that even the existence of the communication is hidden from adversaries. Since traditional encryption methods fall short of providing this level of protection, transmitting data information over wireless open environments like IoT requires the new development of an efficient security approach aware of the privacy feature.

On the other hand, providing reliable communication and high data rate transmission plays an important role in IoT networking developments. This facilitates IoT nodes with limited hardware to achieve stable connectivity and consistent communication quality in the context of dynamic situations such as channel conditions or fading phenomena. Among potential candidates, non-orthogonal multiple access (NOMA) technology is a promising choice for the next generation of IoT networks [5, 6], for its ability to enable multiple NOMA users to share the same time slots, frequency bands, and spreading codes by taking advantages of user signal superimposed in the power domain and successive-interference cancellation technique to separate the signals [7–9]. Accordingly, NOMA has been investigated in various scenarios of wireless transmissions. For example, in millimeter wave multiple-input multiple-output systems, the work in [10] considered finding the optimal random beamforming coefficients by minimizing the outage probability (OP) for NOMA users. The work in [11] proposed the use of space-time block coding schemes combined with NOMA to reduce communication overhead and latency. In [12], the authors employed random near-far NOMA pairing while optimizing the power allocation and beamforming coefficients to enhance the system performance. Another example of NOMA applications is in limited energy resources. For example, the work in [13] investigated the NOMA for cooperative IoT networks in the presence of co-channel interference. In [14], the authors investigated the functionality of NOMA combined with simultaneously transmitting and reflecting intelligent surface, along with analysis of the performance limits of OP and the tradeoffs between energy and rate as well as between energy and reliability.

NOMA not only delivers fundamental benefits as mentioned above but also helps improve security and covert communication. For example, by dynamically managing power distribution and refining coding and decoding methods, NOMA can help reduce the risks of eavesdropping and illegal entities [15–18]. Especially in cases where a transmitter needs to communicate with a receiver without being detected by a warden, NOMA can leverage background noise in conjunction with inter-user interference (i.e., covert signal is encoded together with public or overt signal) to confuse the surveillance activity, thereby improving covert transmission [19–23].

### 1.2 Literature review

#### 1.2.1 Reliability aspect

The popularity of NOMA in improving transmission reliability for IoT networks has been demonstrated in several studies in recent years. For instance, a novel target power allocation (PA) approach for uplink NOMA-based IoT systems was proposed in [24], with an emphasis on the importance of the trade-off between sum rate and reliability in enhancing the overall performance. By generalizing the model proposed in [24] to cellular IoT networks, an adaptive rate NOMA scheme was introduced in [25] to enhance the IoT user capacity and reduce delay transmissions simultaneously. In [26], NOMA protocols were exploited as a bridge to connect cellular systems with IoT networks, allowing the former to have wide communication coverage to support cell-edge users and the latter to flexibly access licensed spectrum to enhance its quality-of-service. The efficacy of this shared communication protocol was confirmed through the analysis of OP. The benefit of the NOMA protocol in improving reliable communication was demonstrated in wirelessly powered cognitive radio paradigms [27], where the authors show that jointly optimizing user power distribution and energy harvesting time-switching factor can efficiently minimize the OP and maximize the system throughput.

#### 1.2.2 Secure aspect

Similar to the reliability aspect, the research on NOMA with physical-layer security has also been explored in many eavesdropper contexts. For example, in [15], a joint power and beamforming design was proposed to deal with the issue of pairing untrusted near users with far users. In [16], the performance quality of terrestrial-integrated aerial IoT NOMA systems with the existence of aerial eavesdroppers was characterized by a secrecy outage probability (SOP) framework. To avoid the outage floor in a secured NOMA system with short-packet transmission, a novel PA strategy was developed in [17], along with analyses of trade-offs in security-efficiency and security-reliability. In [18], the authors analyzed the security performance of short-packet NOMA-IoT networks by deriving a secrecy rate formula.

#### 1.2.3 Covert aspect

Several investigations have analyzed the prospect of NOMA in IoT systems from various covert communication perspectives. In [19], a covert NOMA scheme was introduced for cooperative device-to-device communication. This scheme focuses on characterizing the detection error probability (DEP) of the eavesdropper, followed by the finding of the minimal DEP as the worst-case scenario of the main system to lay the foundation for maximizing covert throughput. Inspired by this, the work in [20] proposed to enhance the covert throughput in downlink NOMA IoT systems by optimizing the PA policy under the constraints of the DEP and OP. Similarly, the work in [21] also focused on the same covert maximization problem in [20] but in light of the network’s uncertain channel state information. In [22], the authors examined the ergodic rate maximization by optimizing the power resource under the detection threshold constraint. The work in [23] designed a random artificial noise-based beamforming scheme to reduce the eavesdropping rate of the strong user while enhancing the covert communication rate. Very recently, the work in [28] presented a comprehensive analysis and optimization frameworks for the OP, SOP, and DEP performance of active reconfigurable repeater for NOMA systems in the context of IoTs.

Despite the promise of NOMA in providing high-reliability transmission, secured communication services, and advanced covert transmission, very limited studies have explored enhancing covert communication quality in NOMA-based IoT systems through a joint assessment of reliability, security, and confidentiality. For example, the research in [15, 16, 18] provides solid mathematical frameworks for SOP, effective secrecy throughput, and effective secrecy rate but their correlation in enhancing the quality of the system has been not touched yet except for [17]. Similarly, the works in [19–23] mostly study how to derive the mathematical frameworks for the OP, DEP, and effective covert throughput, where only two investigations in [20, 21] take into consideration of both DEP and OP constraints to the covert throughput maximization. Likewise, the work in [28] investigated the analysis and optimization tasks for the OP, SOP, and DEP but in separate manners.

### 1.3 Novelty and contributions

As discussed in Sections 1.1 and 1.2, given the potential nature of NOMA technology, it is apparent that the development of joint secure and confidential communication protocols to improve reliable transmission in IoT networks has not yet received sufficient attention. Therefore, increasing research efforts in this aspect is timely and necessary. To fill this important gap in the literature, we focus on jamming-assisted covert NOMA systems, where the covert signal of a nearby user is embedded with a public signal of the far user using NOMA transmission and a friendly jammer is deployed to confuse the surveillance of eavesdroppers. In this context, we start with a mathematical framework for evaluating key performance indicators of reliability, security and covertness to comprehensively observe the system characteristics. Then, we provide an optimization framework to enhance the quality of covert transmission in NOMA-based systems. In summary, the main contributions of this work include

1. From the eavesdropper’s perspective, we derive the exact DEP expression for the surveillance situation. We then implement the lower-bound optimal DEP by considering the worst-case scenario where the eavesdropper can optimize his judgment threshold. This analysis will serve as an effective guideline for developing a covert communication design of the main system. By assuming that the eavesdropper is able to distinguish the covert signal from the overt signal through monitoring, we further quantify the exact and approximate closed-form expressions for the SOP in the eavesdropping situation.
2. From the users’ perspective, we derive closed-form expressions for the covert and public OP as well as ergodic rate formulas. To gain insight into the impact of the transmitted signal-to-noise ratio (SNR) on users’ performance, we have also conducted the asymptotic OP and ergodic rate analyses.
3. Building upon both user and eavesdropper behaviors, we formulate and address two problems of optimizing the NOMA power distribution to minimize covert OP and maximize covert ergodic rate under strict requirements of systems, where sub-optimal closed-form expressions for the PA coefficient are derived. By realizing our proposed optimization frameworks, the system can achieve triple goals simultaneously, including reliability, security, and covertness.

Numerical results verify the correctness of the developed mathematical frameworks while demonstrating the proposed optimization frameworks in minimizing covert outage performance and maximizing the covert rate transmission, both following stringent requirements of reliability, security and covertness.

### 1.4 Structure of the paper

The remaining structure of this work can be summarized as follows. We start with the system model description in Section 2, followed by Section 3 with detailed performance analysis, critical problems on providing effective covert communication design, and how to solve these problems. Section 4 provides numerical examples to validate the developed mathematical evaluation and optimization frameworks on the one hand as well as explore the impacts of system parameters on the other hand. Finally, we conclude the paper with Section 5.

## 2 System model description

**Fig 1:**
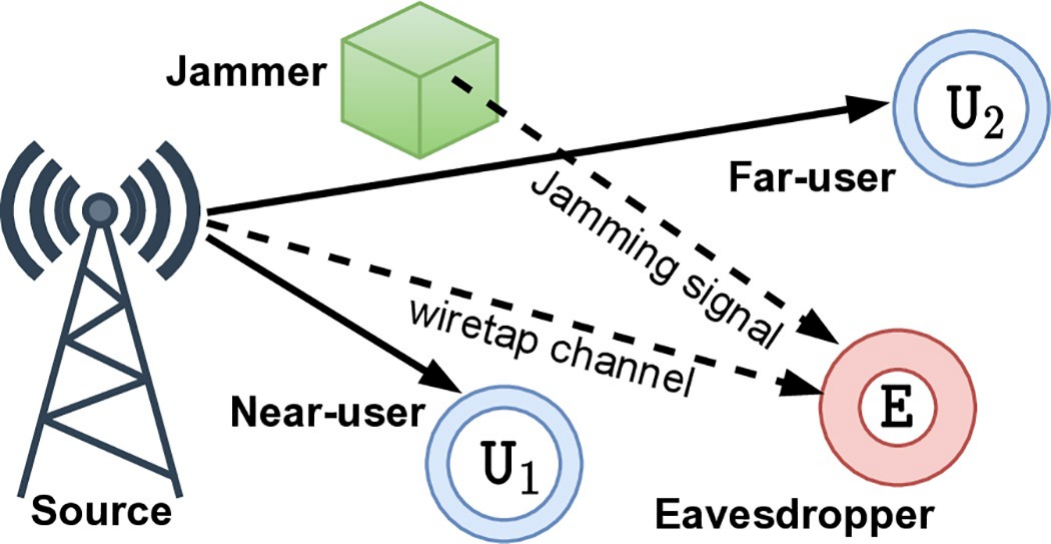
A downlink NOMA system: One source, one jammer, a pair of NOMA users, and one eavesdropper.

As depicted in Fig 1, we consider a downlink NOMA system, where source information S communicates with two users, one covert near user $U_{1}$ and one public far user $U_{2}$, by NOMA signaling $x_{\text{no}}=\sqrt{\rho }x_{2}+\sqrt{\left(1-\rho \right)}x_{1}$ under the surveillance of an eavesdropper E who can be an idle user or potential eavesdropping candidate. 0.5 < *ρ* < 1 is the PA factor while *x*_1_ and *x*_2_ represent the intended signals of $U_{1}$ and $U_{2}$, respectively, such that $E\{|x_{1}\left|\right\}=E\{|x_{2}\left|\right\}=0$ and $E\{|x_{1}{|}^{2}\}=E\{|x_{2}{|}^{2}\}=1$. A jammer (J) is deployed to support main channel communication by continuously generating artificial noise *x*_*ja*_ with power *p*_*j*_ to degrade the quality of E, and *x*_*ja*_ is shared for users, with $E\{|x_{ja}{|}^{2}\}=1$. In this investigation, both $U_{1}$ and $U_{2}$ are assumed to know the channel state information (CSI) from S and J to themselves, enabling them to remove the jamming signal detection from the received signal, then subtract this jamming component, and finally decode their information signal. This CSI can be obtained via uplink pilot training while the absence of channel feedback results in S only obtaining the statistical CSI of E. We focus on the scenario E performs two phases of detection and decoding processes to determine whether S transmits a signal to $U_{2}$ and then goes into decoding $U_{2}$’s signal if it is detected.

Accordingly, to better enhance its physical layer security performance, let us go into the details of communication characteristics at $U_{2}$, $U_{1}$, and E. Denote by $g_{XY}$ is the channel of links from node X∈{S,J} to node $Y\in \{U_{2},U_{1},E\}$, and it follows Rayleigh fading distribution with scale parameter $\Omega_{XY}$. Thus, the Probability Density Functions (PDF) and Cumulative Distribution Functions (CDF) of the channel gain $|g_{XY}{|}^{2}$ can be, respectively, modeled as in [9, Eqs. (13) and (14)] as

$$\begin{array}{c} f_{|g_{XY}{|}^{2}}\left(x\right)=\text{exp}(-x/\Omega_{XY})/\Omega_{XY}, \end{array}$$ (1)

$$\begin{array}{c} F_{|g_{XY}{|}^{2}}\left(x\right)=1-\text{exp}(-x/\Omega_{XY}). \end{array}$$ (2)

Given the transmit power *p*_*s*_ by S, the signal received at Y under the additive white Gaussian noise (AWGN) $n_{Y}$ can be rewritten as

$$\begin{array}{c} y_{Y}=\sqrt{p_{s}}x_{\text{no}}g_{SY}+\sqrt{p_{j}}x_{ja}g_{JY}+n_{Y},\;n_{Y}∼CN\left(0,\sigma^{2}\right). \end{array}$$ (3)

Due to a smaller power level allocated, $U_{1}$ decodes his signal by performing the successive-interference cancellation (SIC) process to subtract the decoded signal *x*_2_ from the received signal and then decode *x*_1_ under perfect SIC assumption. The achievable rates of decoding *x*_2_ and *x*_1_ at $U_{1}$ can be respectively written as

$$\begin{array}{c} C_{U_{1}}^{x_{2}}={\text{log}}_{2}(1+\frac{\rho {\bar{\gamma }}_{s}{\left|h_{{SU}_{1}}\right|}^{2}}{\left(1-\rho \right){\bar{\gamma }}_{s}{\left|h_{{SU}_{1}}\right|}^{2}+1}),C_{U_{1}}^{x_{1}}={\text{log}}_{2}(1+\left(1-\rho \right){\bar{\gamma }}_{s}{\left|h_{{SU}_{1}}\right|}^{2}), \end{array}$$ (4)

where ${\bar{\gamma }}_{s}=p_{s}/\sigma^{2}$ presents the average transmit signal-to-noise ratio (SNR). As an unauthorized entity, E has no information regarding *x*_1_, and thus, a radiometer is employed with two hypotheses to determine the existence of *x*_1_: *h*_1_ (true—the existence of *x*_1_) and *h*_0_ (false—the none of *x*_1_). Thus, the signal observed at E can be classified into

$$\begin{array}{c} y_{E}=\{\begin{array}{cc} \sqrt{p_{s}}x_{\text{no}}h_{SE}+\sqrt{p_{j}}g_{JE}+n_{E}, & h_{1}\\ \sqrt{p_{s}}\sqrt{\rho }x_{2}h_{SE}+\sqrt{p_{j}}g_{JE}+n_{E}, & h_{0} \end{array} \end{array}$$ (5)

Denote by *d*_1_ and *d*_0_ the respective decisions of events *h*_1_ and *h*_0_, and *ζ* is the judgment threshold for deciding whether *h*_1_ or *h*_0_.

Suppose that E is able to successfully detect *x*_1_; and thus, the achievable rate of decoding *x*_1_ at E can be expressed as

$$\begin{array}{c} C_{E}={\text{log}}_{2}(1+\frac{\left(1-\rho \right){\bar{\gamma }}_{s}{\left|g_{SE}\right|}^{2}}{{\bar{\gamma }}_{j}{\left|g_{JE}\right|}^{2}+1}). \end{array}$$ (6)

From the aforementioned above, we next turn to explore the performance behaviour of monitoring and eavesdropping activities and then establish an appropriate strategy to enhance the system’s covert performance in the following section.

## 3 Performance analysis, problem statement, and solution

This section first analyzes the performance of detecting *x*_1_ and intercepting *x*_1_ at E, then formulate the strategies to enhance the system performance, and finally provide the corresponding efficient solutions.

### 3.1 Performance analysis

In the context of performance analysis, achieving closed-form expressions in wireless performance analysis helps provide simplified and exact representations of complex relationships, enabling quick and efficient evaluations of performance metrics, and offering deep insights into the system behaviour without extensive simulations. This is because such closed-form expressions can be readily programmable by common package software like Matlab, mathematical, and Maple. As such, one can evaluate specific scenarios of network deployments by replacing input parameters before using them for online implementations. Therefore, in this study, we will analyze the system performance by first deriving closed-form expressions for the DEP, SOP, OP, and rate metrics.

#### 3.1.1 Monitoring analysis

To have the best knowledge on the behaviour of monitoring *x*_1_ at E for improving the security transmission at the physical layer, this subsection will analyze the DEP in the role of an eavesdropper. This is because the DEP metric will reflect the level of error probability that the eavesdropper encounters during the monitoring process. To be specific, we first derive the DEP by assuming the judgment threshold is fixed, i.e., *ζ*. Based on this, we then quantify how the eavesdropper will optimize the judgment threshold *ζ* to minimize the DEP metric. Such analysis would provide useful benchmarks in enhancing security countermeasures since it focuses on the worst-case scenario, where DEP is minimized at an eavesdropper.

In detail, we first go into deriving the exact DEP, which can be mathematically written as

$$\begin{array}{c} {\text{DEP}}_{E}=\text{Pr}\left[d_{1}|h_{0}\right]+\text{Pr}\left[d_{0}|h_{1}\right], \end{array}$$ (7)

where the first probability implies that the transmission of *x*_1_ observed by E exists but there is no actual transmission. In contrast, the second probability refers to no transmission observed by E, but there is a transmission of *x*_1_.

Similar to [19–23], we assume that the infinite number of signal examples are collected by E to detect *x*_2_. Thus, the first probability in (7) can be derived from (5) as

$$\begin{array}{cc} \text{Pr}[d_{1}|H_{0}] & =\text{Pr}[p_{s}\rho |h_{SE}{|}^{2}+p_{j}{\left|h_{JE}\right|}^{2}+\sigma^{2}\geq \zeta ]=1-\text{Pr}[{\bar{\gamma }}_{s}\rho |h_{SE}{|}^{2}\leq \Delta -{\bar{\gamma }}_{j}{\left|h_{JE}\right|}^{2}]\\ & =1-\int_{0}^{\frac{\Delta }{{\bar{\gamma }}_{j}}}F_{|h_{SE}{|}^{2}}(\frac{\Delta -{\bar{\gamma }}_{j}x}{{\bar{\gamma }}_{s}\rho })f_{|h_{JE}{|}^{2}}\left(x\right)dx\\ & =1-F_{|h_{JE}{|}^{2}}(\frac{\Delta }{{\bar{\gamma }}_{j}})+\int_{0}^{\Delta /{\bar{\gamma }}_{j}}\text{exp}(-\frac{x}{\Omega_{JE}})\frac{1}{\Omega_{JE}}\text{exp}(-\frac{\Delta -{\bar{\gamma }}_{j}x}{{\bar{\gamma }}_{s}\rho \Omega_{SE}})dx\\ & =1-F_{|h_{JE}{|}^{2}}(\frac{\Delta }{{\bar{\gamma }}_{j}})+\frac{{\bar{\gamma }}_{s}\rho \Omega_{SE}}{{\bar{\gamma }}_{s}\rho \Omega_{SE}-{\bar{\gamma }}_{j}\Omega_{JE}}[\text{exp}(-\frac{\Delta }{{\bar{\gamma }}_{s}\rho \Omega_{SE}})-\text{exp}(-\frac{\Delta }{{\bar{\gamma }}_{j}\Omega_{JE}})]. \end{array}$$ (8)

where ${\bar{\gamma }}_{j}=p_{j}/\sigma^{2}$ is the jammer SNR and Δ = *ζ*/*σ*^2^ − 1. Meanwhile, the second probability in (7) can be derived as

$$\begin{array}{cc} \text{Pr}[d_{0}|H_{1}] & =\text{Pr}[p_{s}|h_{SE}{|}^{2}+p_{j}{\left|h_{JE}\right|}^{2}+\sigma^{2}\leq \zeta ]\\ & =F_{|h_{JE}{|}^{2}}\left(\Delta /{\bar{\gamma }}_{j}\right)-\frac{{\bar{\gamma }}_{s}\Omega_{SE}}{{\bar{\gamma }}_{s}\Omega_{SE}-{\bar{\gamma }}_{j}\Omega_{JE}}[\text{exp}(-\frac{\Delta }{{\bar{\gamma }}_{s}\Omega_{SE}})-\text{exp}(-\frac{\Delta }{{\bar{\gamma }}_{j}\Omega_{JE}})]. \end{array}$$ (9)

By plugging (8) and (9) into (7), we get the following theorem.

**Lemma 1**. The closed-form expression for the DEP of E can be derived as

$$\begin{array}{cc} {\text{DEP}}_{E}=1 & +\frac{\text{exp}(-\frac{\Delta }{{\bar{\gamma }}_{s}\rho \Omega_{SE}})}{1-\frac{{\bar{\gamma }}_{j}\Omega_{JE}}{{\bar{\gamma }}_{s}\rho \Omega_{SE}}}-\frac{\text{exp}(-\frac{\Delta }{{\bar{\gamma }}_{s}\Omega_{SE}})}{1-\frac{{\bar{\gamma }}_{j}\Omega_{JE}}{{\bar{\gamma }}_{s}\Omega_{SE}}}+\frac{1}{1-\frac{{\bar{\gamma }}_{j}\Omega_{JE}}{{\bar{\gamma }}_{s}\Omega_{SE}}}\frac{\left(1-\rho \right)}{1-\frac{\rho {\bar{\gamma }}_{s}\Omega_{SE}}{{\bar{\gamma }}_{j}\Omega_{JE}}}\text{exp}(-\frac{\Delta }{{\bar{\gamma }}_{j}\Omega_{JE}}). \end{array}$$ (10)

The result in (10) shows that *σ*^2^, $\Omega_{SE}$, *ζ*, and $\Omega_{JE}$ are arbitrary values without controlling ability. Meanwhile, examining ${\bar{\gamma }}_{s}$ and ${\bar{\gamma }}_{j}$ discloses that when ${\bar{\gamma }}_{s},{\bar{\gamma }}_{j}\to \infty$ occurs, the DEP ${\text{DEP}}_{E}^{⋆}$ at E becomes unity, i.e., ${\text{DEP}}_{E}\to 1$. This means that ${\text{DEP}}_{E}$ is an increasing function of ${\bar{\gamma }}_{s},{\bar{\gamma }}_{j}\to \infty$. However, this is only correct when E does not optimize its judgement threshold *ζ*. Therefore, deriving the optimal DEP at E with the optimization of *ζ*^⋆^ is necessary but not easy, which motivates us to introduce the following lower-bound optimal DEP.

**Proposition 1**. The lower-bound optimal DEP at E can be derived as

$$\begin{array}{c} \tilde{{\text{DEP}}_{E}^{⋆}}=\rho^{\frac{1}{\left(1-\rho \right)}}+1-\rho^{\frac{\rho }{\left(1-\rho \right)}}. \end{array}$$ (11)

*Proof*. Let us reconsider the probabilities in (7) by conditioning on $X=|h_{JE}{|}^{2}$ as

$$\begin{array}{c} \text{Pr}[d_{1}\left|H_{0}\right|X]=1-\text{Pr}[p_{s}\rho {\left|h_{SE}\right|}^{2}\leq \zeta -p_{j}X-\sigma^{2}]=\text{exp}(-\frac{\Delta -{\bar{\gamma }}_{j}X}{\Omega_{SE}{\bar{\gamma }}_{s}\rho }), \end{array}$$ (12)

$$\begin{array}{c} \text{Pr}[d_{0}\left|H_{1}\right|X]=\text{Pr}[p_{s}|h_{SE}{|}^{2}+p_{j}X+\sigma^{2}\leq \zeta |]=1-\text{exp}(-\frac{\Delta -{\bar{\gamma }}_{j}X}{\Omega_{SE}{\bar{\gamma }}_{s}}). \end{array}$$ (13)

By plugging (12) and (13) into (7) and then taking Δ with respect to *ζ* equal zero, we get that

$$\begin{array}{cc} \frac{\partial {\text{DEP}}_{E}}{\partial \Delta } & =-\frac{1}{\Omega_{SE}{\bar{\gamma }}_{s}\rho }\text{exp}(-\frac{\Delta -{\bar{\gamma }}_{j}X}{\Omega_{SE}{\bar{\gamma }}_{s}\rho })+\frac{1}{\Omega_{SE}{\bar{\gamma }}_{s}}\text{exp}(-\frac{\Delta -{\bar{\gamma }}_{j}X}{\Omega_{SE}{\bar{\gamma }}_{s}})=0\\ \Rightarrow & \frac{1}{\rho }\text{exp}(-\frac{\Delta -{\bar{\gamma }}_{j}X}{\Omega_{SE}{\bar{\gamma }}_{s}\rho })=\text{exp}(-\frac{\Delta -{\bar{\gamma }}_{j}X}{\Omega_{SE}{\bar{\gamma }}_{s}})\\ \Rightarrow & \text{ln}(\frac{1}{\rho })-\frac{\Delta -{\bar{\gamma }}_{j}X}{\Omega_{SE}{\bar{\gamma }}_{s}\rho }=\frac{{\bar{\gamma }}_{j}X-\Delta }{\Omega_{SE}{\bar{\gamma }}_{s}}\\ \Rightarrow & \frac{\Omega_{SE}{\bar{\gamma }}_{s}\rho }{\left(1-\rho \right)}\text{ln}(\frac{1}{\rho })+{\bar{\gamma }}_{j}X=\Delta . \end{array}$$ (14)

Finally, plugging Δ in (12) and (13), we get the lower-bound optimal DEP.

**Remark 1**. The result in (11) reveals that $\tilde{{\text{DEP}}_{E}^{⋆}}$ is increased with an increase in *ρ*. Specifically, when *ρ* = 0, we get $\tilde{{\text{DEP}}_{E}^{⋆}}=0$. When *ρ* = 0.5, $\tilde{{\text{DEP}}_{E}^{⋆}}=1+0.5^{1/0.5}-0.5^{1}=0.75$. When *ρ* → 1, $\tilde{{\text{DEP}}_{E}^{⋆}}\to 1$ since lim_*ρ*→1_ *ρ*^1/(1−*ρ*)^ = 1/exp(1) and lim_*ρ*→1_ *ρ*^*ρ*/(1−*ρ*)^ = 1/exp(1), which is attained based on the property of the exponential function limit. On the other hand, it is worth noting that the result (11) will converge to the exact optimal DEP at E whenever ${\bar{\gamma }}_{j}\leq {\bar{\gamma }}_{s}$, which shall be soon demonstrated in numerical results.

#### 3.1.2 Analysis of eavesdropping activity

In this section, we will further go into a detailed analysis of the case where the eavesdropper can detect the covert signal from the monitoring activity and then try to wiretap this signal. Herein, we focus on evaluating the SOP performance as the role of the legitimate system rather than the eavesdropper in the previous section. Specifically, the SOP metric will reflect how much probability that the legitimate link’s channel capacity $C_{E}$ is larger than the eavesdropper link’s channel capacity $C_{U_{1}}^{x_{1}}$ when compared to a given secrecy rate *R*_1_ of decoding design, i.e., $C_{s}^{x_{1}}=[C_{U_{1}}^{x_{1}}-C_{E}]<R_{1}$. If $C_{U_{1}}^{x_{1}}-C_{E}$ is negative and its magnitude is larger than the secure rate *R*_1_, it means that the eavesdropper can strongly wiretap the covert signal with the probability of one. This means that using security techniques like encryption is not effective. Conversely, $C_{U_{1}}^{x_{1}}-C_{E}$ is positive. This means that the eavesdropper can have a quality of achievable rate worse than the legitimate user, and if this achieved rate gap is larger than the secure rate, the eavesdropper cannot decode any information. In other words, using security techniques is entirely effective.

Following that, the SOP that E eavesdrops on *x*_1_ can be mathematically written as follows:

$$\begin{array}{c} {\text{SOP}}_{E}=\text{Pr}(C_{U_{1}}^{x_{1}}-C_{E}<R_{1}). \end{array}$$ (15)

By substituting (4) and (6) into (15), the SOP that E eavesdrops on *x*_1_ can be derived as

$$\begin{array}{cc} {\text{SOP}}_{E} & =\text{Pr}(\frac{1+\left(1-\rho \right){\bar{\gamma }}_{s}{\left|g_{{SU}_{1}}\right|}^{2}}{1+\frac{\left(1-\rho \right){\bar{\gamma }}_{s}{\left|g_{SE}\right|}^{2}}{Y}}<\tau |Y≜{\bar{\gamma }}_{j}{\left|g_{JE}\right|}^{2}+1)\\ & =\int_{0}^{\infty }F_{|g_{{SU}_{1}}{|}^{2}}(\frac{\phi +\tau \left(1-\rho \right){\bar{\gamma }}_{s}x/Y}{\left(1-\rho \right){\bar{\gamma }}_{s}})f_{|g_{SE}{|}^{2}}\left(x\right)dx\\ & =1-\text{exp}(-\frac{\phi /\Omega_{{SU}_{1}}}{\left(1-\rho \right){\bar{\gamma }}_{s}})\int_{1}^{\infty }\frac{yf_{Y}\left(y\right)dy}{y+\tau \Omega_{SE}/\Omega_{{SU}_{1}}}\\ & =1-\text{exp}(-\frac{\phi }{\left(1-\rho \right){\bar{\gamma }}_{s}\Omega_{{SU}_{1}}}+\frac{1}{{\bar{\gamma }}_{j}\Omega_{JE}})\int_{1}^{\infty }\frac{y\text{exp}(-\frac{x}{{\bar{\gamma }}_{j}\Omega_{JE}})}{y+\tau \Omega_{SE}/\Omega_{{SU}_{1}}}\frac{1}{{\bar{\gamma }}_{j}\Omega_{JE}}dx\\ & =1-\text{exp}(-\frac{\phi }{\left(1-\rho \right){\bar{\gamma }}_{s}\Omega_{{SU}_{1}}}+\frac{1}{{\bar{\gamma }}_{j}\Omega_{JE}})\frac{1}{{\bar{\gamma }}_{j}\Omega_{JE}}\\ & \;\times \int_{1}^{\infty }[1-\frac{\tau \Omega_{SE}/\Omega_{{SU}_{1}}}{y+\tau \Omega_{SE}/\Omega_{{SU}_{1}}}]\text{exp}(-\frac{x}{{\bar{\gamma }}_{j}\Omega_{JE}})dx, \end{array}$$ (16)

where $\phi =2^{R_{1}}-1$. Making use of [29, eq. (3.352.2)], we achieve the following lemma.

**Lemma 2**. Exact closed-form expressions for the SOP that E eavesdrops on *x*_1_ can be derived as

$$\begin{array}{cc} & {\text{SOP}}_{E}=1-\text{exp}(-\phi /[\left(1-\rho \right){\bar{\gamma }}_{s}\Omega_{{SU}_{1}}])\Xi , \end{array}$$ (17)

where Ξ is the short-notation of

$$\Xi =1+\frac{\tau \Omega_{SE}}{\Omega_{{SU}_{1}}{\bar{\gamma }}_{j}\Omega_{JE}}\text{exp}(-\frac{1}{{\bar{\gamma }}_{j}\Omega_{JE}}+\frac{\tau \Omega_{SE}}{\Omega_{{SU}_{1}}{\bar{\gamma }}_{j}\Omega_{JE}})\text{Ei}(-\frac{1}{{\bar{\gamma }}_{j}\Omega_{JE}}-\frac{\tau \Omega_{SE}}{\Omega_{{SU}_{1}}{\bar{\gamma }}_{j}\Omega_{JE}}),$$ (18)

where Ei(⋅) is the exponential integral function [29, Eq. (8.211.1)]. This function is special in mathematics and is defined as the integral of the ratio between an exponential function and its argument. For real non-zero values of (*x*), the exponential integral is given by:

$$\begin{array}{c} \text{Ei}\left(x\right)=-\int_{-x}^{\infty }\frac{e^{-t}}{t}dt=\int_{-\infty }^{x}\frac{e^{t}}{t}dt. \end{array}$$ (19)

**Remark 2**. As high SNR, i.e., ${\bar{\gamma }}_{s}\to \infty$, the SOP of $U_{1}$ in (17) can be upper bounded using the connection 1 − exp(−*x*) ≃ *x* as *x* → 0 to get that

$$\begin{array}{cc} & \tilde{{\text{SOP}}_{E}}=1-(1-\phi /[\left(1-\rho \right){\bar{\gamma }}_{s}\Omega_{{SU}_{1}}])\Xi . \end{array}$$ (20)

**Remark 3**. The result in (20) reveals that the SOP at E is an increasing function of *ρ* since exp(−1/(1 − *ρ*)) → 0. When ${\bar{\gamma }}_{s}\to \infty$, the SOP at E becomes ${\text{SOP}}_{E}≃\Xi$ (a constant value).

#### 3.1.3 Analysis of legitimate user

This part evaluates the performance of $U_{1}$ by deriving the OP and ergodic rate metric. Herein, the OP metric will reflect how much reliable communication users can achieve with the given predefined threshold rate, respectively. Meanwhile, the ergodic rate will clarify how much the average achievable rate that users can obtain when the system uses channel coding/modulation schemes to achieve near-zero errors during signal transmission.

**OP analysis for** $U_{1}$ : Let *r*_*i*_ is the given predefined threshold rate to decode *x*_*i*_, with *i* = 1, 2. Denoting $\kappa_{i}=2^{r_{i}}-1$, the OP of decoding *x*_1_ at $U_{1}$ can be mathematically defined as

$$\begin{array}{c} {\text{OP}}_{U_{1}}=1-\text{Pr}\left[C_{U_{1}}^{x_{2}}>r_{2},C_{U_{1}}^{x_{1}}>r_{1}\right]. \end{array}$$ (21)

This probability describes all the events that the achievable rates received by $U_{1}$ for decoding *x*_1_ or *x*_2_ or both of them are much less than the required target rates. This means that $U_{1}$ cannot decode either *x*_1_ or *x*_2_ or both of them.

By injecting the formulas of $C_{U_{1}}^{x_{2}}$ and $C_{U_{1}}^{x_{1}}$ in (4) into the above probability, we can rewrite the term ${\text{OP}}_{U_{1}}$ as

$$\begin{array}{cc} {\text{OP}}_{U_{1}} & =1-\text{Pr}[|g_{{SU}_{1}}{|}^{2}>\frac{\kappa_{2}}{{\bar{\gamma }}_{s}\psi },{\left|g_{{SU}_{1}}\right|}^{2}>\frac{\kappa_{1}}{{\bar{\gamma }}_{s}\left(1-\rho \right)}]\\ & =F_{|g_{{SU}_{1}}{|}^{2}}(\frac{1}{{\bar{\gamma }}_{s}}\text{max}\{\frac{\kappa_{2}}{\psi },\frac{\kappa_{1}}{\left(1-\rho \right)}\}), \end{array}$$ (22)

where *ψ* = *ρ* − (1 − *ρ*)*κ*_2_ > 0. Herein, if *ψ* ≤ 0, ${\text{OP}}_{U_{1}}=1$.

By mapping the CDF in (1) with the above result, we obtain the final OP expression for $U_{1}$ as

$$\begin{array}{cc} {\text{OP}}_{U_{1}} & =\{\begin{array}{cc} 1-\text{exp}(-\frac{\text{max}\{\frac{\kappa_{2}}{\psi },\frac{\kappa_{1}}{\left(1-\rho \right)}\}}{{\bar{\gamma }}_{s}\Omega_{{SU}_{1}}}), & \psi <0,\\ 1, & \psi \leq 0. \end{array} \end{array}$$ (23)

**Remark 4**. The result in (23) shows that when *κ*_2_/*ψ* ≥ *κ*_1_/(1 − *ρ*), the effective region of *ρ* is

$$\begin{array}{c} \rho \leq \kappa_{2}\left(1+\kappa_{1}\right)/(\kappa_{1}\left(1+\kappa_{2}\right)+\kappa_{2})≜\varpi . \end{array}$$ (24)

As for this case, increasing *ρ* decreases ${\text{OP}}_{U_{1}}$. Conversely, increasing *ρ* gives rise to ${\text{OP}}_{U_{1}}$.

**Remark 5**. At high SNR, i.e., ${\bar{\gamma }}_{s}\to \infty$, the OP of $U_{1}$ in (23) can be upper bounded as

$$\begin{array}{c} \tilde{{\text{OP}}_{U_{1}}}\approx \text{max}\{\kappa_{2}/(\rho -\left(1-\rho \right)\kappa_{2}),\kappa_{1}\left(/1-\rho \right)\}/\left[{\bar{\gamma }}_{s}\Omega_{{SU}_{1}}\right]. \end{array}$$ (25)

It is clear that $\tilde{{\text{OP}}_{U_{1}}}$ is proportional to $1/{\bar{\gamma }}_{s}$; thus, we can infer that $U_{1}$’s diversity order is 1.

**Covert rate analysis for** $U_{1}$ : The covert rate of sending *x*_1_ from S to $U_{1}$ under no error decoding of *x*_1_ can be defined as

$$R_{U_{1}}=E\{{\text{log}}_{2}(1+\left(1-\rho \right){\bar{\gamma }}_{s}{\left|h_{{SU}_{1}}\right|}^{2})\}.$$ (26)

By applying integral by part methods [13, Eq. (30)] and mapping the CDF in (1), we can rewrite the term $R_{U_{1}}$ as

$$\begin{array}{cc} R_{U_{1}} & =\frac{1}{\text{ln}(2)}\int_{0}^{\infty }\frac{1}{1+x}[1-F_{|h_{{SU}_{1}}{|}^{2}}(\frac{x/{\bar{\gamma }}_{s}}{\left(1-\rho \right)})]dx\\ & =\int_{0}^{\infty }\frac{\text{exp}(-x/[\Omega_{{SU}_{1}}\left(1-\rho \right){\bar{\gamma }}_{s}])}{\text{ln}(2)(1+x)}dx. \end{array}$$ (27)

Now, applying the standard form in [29, Eq. (3.352.4)], we get the final expression for the covert rate of sending *x*_1_ from S to $U_{1}$ as

$$\begin{array}{c} R_{U_{1}}=-\frac{\text{exp}(\frac{1/(1-\rho )}{\Omega_{{SU}_{1}}{\bar{\gamma }}_{s}})}{\text{ln}(2)}\text{Ei}(-\frac{1/(1-\rho )}{\Omega_{{SU}_{1}}{\bar{\gamma }}_{s}}). \end{array}$$ (28)

**Remark 6**. At high SNR, i.e., ${\bar{\gamma }}_{s}\to \infty$, the covert rate of $U_{1}$ in (26) can be upper bounded as

$$\tilde{R_{U_{1}}}\approx E\{{\text{log}}_{2}(\left(1-\rho \right){\bar{\gamma }}_{s}{\left|h_{{SU}_{1}}\right|}^{2})\}={\text{log}}_{2}((1-\rho ){\bar{\gamma }}_{s})+E\{{\text{log}}_{2}(|h_{{SU}_{1}}{|}^{2})\}.$$ (29)

Since $\tilde{R_{U_{1}}}$ scales up with a logarithm function of ${\bar{\gamma }}_{s}$, $U_{1}$’s multiplexing gain is 1. Besides, we can also observe that $\tilde{R_{U_{1}}}$ scales down with an increase in *ρ*.

**OP analysis for** $U_{2}$ : Besides investigating the performance of $U_{1}$, it is also necessary to study the performance of serving $U_{2}$. Specifically, the OP of $U_{2}$ to directly decode its message *x*_2_ can be described as

$$\begin{array}{c} {\text{OP}}_{U_{2}}=\text{Pr}\left[C_{U_{2}}^{x_{2}}<r_{2}\right], \end{array}$$ (30)

where $C_{U_{2}}^{x_{2}}$ can be derived similar to $C_{U_{1}}^{x_{2}}$ in (4) by simply replacing $|g_{{SU}_{1}}{|}^{2}$ with $|g_{{SU}_{2}}{|}^{2}$. Accordingly, the OP of $U_{2}$ can be derived by mapping with the CDF in (1) as

$$\begin{array}{cc} {\text{OP}}_{U_{2}} & =\text{Pr}(|g_{{SU}_{2}}{|}^{2}<\frac{\kappa_{2}}{{\bar{\gamma }}_{s}\psi })=F_{|g_{{SU}_{2}}{|}^{2}}(\frac{\kappa_{2}}{{\bar{\gamma }}_{s}\psi })\\ & =1-\text{exp}(-\frac{\kappa_{2}/\left[\Omega_{{SU}_{2}}{\bar{\gamma }}_{s}\right]}{\left(\rho \left(1+\kappa_{2}\right)-\kappa_{2}\right)}). \end{array}$$ (31)

**Remark 7**. Directly inspection of (31) shows that increasing ${\bar{\gamma }}_{s}$, *ρ* or decreasing *κ*_2_ will decrease ${\text{OP}}_{U_{2}}$, i.e., improving the outage event to the smallest possible extent.

**Remark 8**. At high SNR, i.e., ${\bar{\gamma }}_{s}\to \infty$, the OP of $U_{2}$ can be upper bounded as

$$\begin{array}{c} \tilde{{\text{OP}}_{U_{2}}}\approx \kappa_{2}/\left[{\bar{\gamma }}_{s}\Omega_{{SU}_{1}}\left(\rho -\left(1-\rho \right)\kappa_{2}\right)\right]=\kappa_{2}/\left[{\bar{\gamma }}_{s}\Omega_{{SU}_{1}}\left(\rho \left(1+\kappa_{2}\right)-\kappa_{2}\right)\right]. \end{array}$$ (32)

Since $\tilde{{\text{OP}}_{U_{2}}}$ is proportional to $1/{\bar{\gamma }}_{s}$, $U_{2}$’s diversity gain is 1.

**Covert rate analysis for** $U_{2}$ : The public rate of sending *x*_2_ from S to $U_{2}$ can be defined as

$$\begin{array}{c} R_{U_{2}}=E\{{\text{log}}_{2}(1+\frac{\rho {\bar{\gamma }}_{s}{\left|g_{{SU}_{2}}\right|}^{2}}{\left(1-\rho \right){\bar{\gamma }}_{s}{\left|g_{{SU}_{2}}\right|}^{2}+1})\}, \end{array}$$ (33)

To solve the above expression, we first apply the logarithm decomposition and then apply the integral by part methods as

$$\begin{array}{cc} R_{U_{2}} & =E\{{\text{log}}_{2}(1+{\bar{\gamma }}_{s}{\left|g_{{SU}_{2}}\right|}^{2})\}-E\{{\text{log}}_{2}(1+\left(1-\rho \right){\bar{\gamma }}_{s}{\left|g_{{SU}_{2}}\right|}^{2})\}\\ & =\frac{1}{\text{ln}(2)}\int_{0}^{\infty }\frac{1-F_{|h_{{SU}_{2}}{|}^{2}}(\frac{x}{{\bar{\gamma }}_{s}})}{1+x}dx-\frac{1}{\text{ln}(2)}\int_{0}^{\infty }\frac{1-F_{|h_{{SU}_{2}}{|}^{2}}(\frac{x}{\left(1-\rho \right){\bar{\gamma }}_{s}})}{1+x}dx. \end{array}$$ (34)

Now, using the standard form in [29, Eq. (3.352.4)], we get the final expression for the public rate of sending *x*_2_ from S to $U_{2}$ as

$$R_{U_{2}}=\frac{\text{Ei}(-1/[\left(1-\rho \right)\Omega_{{SU}_{2}}{\bar{\gamma }}_{s}])}{\text{ln}\left(2\right)\text{exp}(-1/\left[\left(1-\rho \right)\Omega_{{SU}_{2}}{\bar{\gamma }}_{s}\right])}-\frac{\text{Ei}(-1/\left[\Omega_{{SU}_{2}}{\bar{\gamma }}_{s}\right])}{\text{ln}\left(2\right)\text{exp}(-1/[\Omega_{{SU}_{2}}{\bar{\gamma }}_{s}])}.$$ (35)

**Remark 9**. At high SNR, i.e., ${\bar{\gamma }}_{s}\to \infty$, the public rate of $U_{2}$ can be upper bounded as

$$\tilde{R_{U_{2}}}\approx E\{{\text{log}}_{2}({\bar{\gamma }}_{s}|g_{{SU}_{2}}{|}^{2})\}-E\{{\text{log}}_{2}(\left(1-\rho \right){\bar{\gamma }}_{s}{\left|g_{{SU}_{2}}\right|}^{2})\}=-{\text{log}}_{2}(1-\rho ).$$ (36)

Since $\tilde{R_{U_{2}}}$ does not scale up with a logarithm function of ${\bar{\gamma }}_{s}$, $U_{2}$’s multiplexing gain is 0. Besides, we can also observe that $\tilde{R_{U_{1}}}$ scales up with an increase in *ρ*.

### 3.2 Outage performance optimization

Having all the expressions of the lower-bound optimal DEP, the SOP, and the OP metrics in hand, in this section, we will first formulate the problem of optimizing resource power allocation subject to minimize the reliability of sending covert information while ensuring reliable communication of public signal at all legitimate users (passing SIC process at $U_{1}$ and successfully decoding at $U_{2}$) as well as two lines of security countermeasures for the physical layer transmission (one line from the monitoring signal of covert signals and another line for decoding covert information). Afterward, we will provide detailed guidance in achieving closed-form sub-optimal PA policy.

#### 3.2.1 Problem formulation

In this subsection, we seek an efficient approach to ensure the covert transmission is not disclosed by an unauthorized thirsty party E while ensuring reliable communication for all users. To be specific, we aim to improve the covert transmission of $U_{1}$ in terms of OP by optimizing the PA coefficient *ρ* while ensuring the maximum eavesdropping $\epsilon_{E}$, the minimum covertness $\varrho_{U_{2}}$, and the minimum OP $\varepsilon_{U_{2}}$. Mathematically, this optimization problem can be formulated as

$$\begin{array}{cc} \; & \;\underset{\rho }{\text{min}}\;{\text{OP}}_{U_{1}} \end{array}$$ (37a)

$$\text{s.t}\;\;{\text{OP}}_{U_{2}}\leq \varepsilon_{U_{2}},{\text{SOP}}_{E}\leq \epsilon_{E},{\text{DEP}}_{E}^{⋆}\geq \varrho_{U_{2}},$$ (37b)

$$\;\rho \geq \kappa_{2}/(1+\kappa_{2})+\ell ,1-\ell \geq \rho \geq 0.5+\ell ,\ell ≃0,$$ (37c)

where constraints in (37b) refers to the minimal OP of $U_{2}$, the minimal SOP of E, and the minimal DEP of E, while constraints in (37c) is the operating condition.

#### 3.2.2 Solution approach

Since the problem in (37) involves the complicated expression ${\text{DEP}}_{E}^{⋆}$, finding the exact optimal PA solution *ρ*^⋆^ is an extremely intricate task. We therefore overcome this challenge by leveraging the connection $\tilde{{\text{DEP}}_{E}^{⋆}}\leq {\text{DEP}}_{E}^{⋆}$. This enables us to simplify the optimization problem in (37) into a tractable problem by checking the feasible domain of *ρ*.

Specifically, we begin with revisiting **Remarks** 1, 3, and 7, where we can obtain: $\tilde{{\text{DEP}}_{E}^{⋆}}$ and ${\text{SOP}}_{E}$ are an increasing function of *ρ*, respectively, while ${\text{OP}}_{U_{2}}$ is a decreasing function of *ρ*. Based on this, we solve ${\text{SOP}}_{E}\leq \epsilon_{E}$ to get the second feasible region as

$$\begin{array}{c} \rho \leq \underset{≜\;\theta }{\underset{︸}{1+\frac{\phi }{{\bar{\gamma }}_{s}\Omega_{{SU}_{1}}\text{ln}(\frac{1-\epsilon_{E}}{\Xi })}}}, \end{array}$$ (38)

and solve $\tilde{{\text{DEP}}_{E}^{⋆}}\geq \varrho_{U_{2}}$ to get the following inequality

$$\begin{array}{c} f\left(\rho \right)≜\rho^{1/(1-\rho )}-\rho^{\rho /(1-\rho )}+1-\varrho_{U_{2}}\geq 0. \end{array}$$ (39)

However, finding the exact solution for (39) is extremely difficult; thus, we rely on Newton’s method for finding a root *ρ*^+^ of a function *f*(*ρ*) = 0, which has derivative

$$\begin{array}{c} f^{\prime }\left(\rho \right)=\text{ln}(\rho )\rho^{\frac{\rho }{1-\rho }}/(\rho -1). \end{array}$$ (40)

Thus, the fourth feasible region for (39) can be deduced as

$$\begin{array}{c} \rho \geq \rho^{+}. \end{array}$$ (41)

Next, we solve ${\text{OP}}_{U_{2}}\leq \varepsilon_{U_{2}}$ to have the following inequality

$$\begin{array}{c} \rho \geq \underset{≜\;\varkappa }{\underset{︸}{\frac{\kappa_{2}}{\left(1+\kappa_{2}\right)}-\frac{\kappa_{2}}{\left(1+\kappa_{2}\right)\Omega_{{SU}_{2}}{\bar{\gamma }}_{s}\text{ln}(1-\varepsilon_{U_{2}})}}}. \end{array}$$ (42)

To proceed, we move on exploring the objective function ${\text{OP}}_{U_{1}}$ in (37a) and we notice that it is only improved if (24) holds. Combining this with (37c), we get the feasible region

$$\begin{array}{c} \varphi \leq \rho \leq \xi , \end{array}$$ (43)

where *φ* ≜ max{*κ*_2_/(1 + *κ*_2_) + *ℓ*, 0.5 + *ℓ*} and *ξ* ≜ min{*κ*_2_(1 + *κ*_1_)/(*κ*_1_(1 + *κ*_2_) + *κ*_2_), 1 − *ℓ*}. Putting (38) and (41)–(43) together, we obtain the sub-optimal solution for (37) as

$$\begin{array}{c} \;\rho^{⋆}=\{\begin{array}{cc} \text{min}\{\theta ,\xi \}, & \text{max}\left\{\varphi ,\rho^{+},\varkappa \right\}\leq \text{min}\left\{\theta ,\xi \right\},\\ \text{No}\;\text{solution}, & \text{max}\left\{\varphi ,\rho^{+},\varkappa \right\}>\text{min}\left\{\theta ,\xi \right\}. \end{array} \end{array}$$ (44)

### 3.3 Covert rate maximization

Having all the expressions of the lower-bound optimal DEP, the SOP, and the ergodic rate metrics in hand, in this section, we will first formulate the problem of optimizing resource power allocation subject to maximize the covert rate of sending covert information while ensuring reliable communication of public signal at all legitimate users as well as two lines of security countermeasures for the physical layer transmission. Afterward, we will provide detailed guidance in achieving closed-form sub-optimal PA policy.

#### 3.3.1 Problem formulation

This section aims to optimize *ρ* to maximize the covert rate of $U_{1}$ while ensuring the systems’ security and covert requirements. Specifically, the optimization problem can be formulated as

$$\begin{array}{cc} \; & \;\underset{\rho }{\text{max}}\;R_{U_{1}} \end{array}$$ (45a)

$$\text{s.t}\;\;{\text{SOP}}_{E}\leq \epsilon_{E},{\text{DEP}}_{E}^{⋆}\geq \varrho_{U_{2}},$$ (45b)

$$\;{\text{OP}}_{U_{1}}\leq \varepsilon_{U_{1}},{\text{OP}}_{U_{2}}\leq \varepsilon_{U_{2}},$$ (45c)

$$\;\rho \geq \kappa_{2}/(1+\kappa_{2})+\ell ,1-\ell \geq \rho \geq 0.5+\ell ,\ell ≃0,$$ (45d)

where $\varepsilon_{U_{1}}$ is the minimum OP required for $U_{1}$.

#### 3.3.2 Solution approach

Since the complex results of (45a) and ${\text{DEP}}_{E}^{⋆}\geq \varrho_{U_{2}}$ in (45d) makes obtaining the optimal solution for the problem in (45), we tackle this problem by leveraging the connection $\tilde{{\text{DEP}}_{E}^{⋆}}\leq {\text{DEP}}_{E}^{⋆}$, providing the feasible region in (42). Next, we solve ${\text{SOP}}_{E}\leq \epsilon_{E}$ and ${\text{OP}}_{U_{2}}\leq \varepsilon_{U_{2}}$, returning the respective results in (38) and (43). Meanwhile, solving ${\text{OP}}_{U_{1}}\leq \varepsilon_{U_{1}}$ results in

$$\begin{array}{cc} & 1-\text{exp}(-\frac{1}{{\bar{\gamma }}_{s}\Omega_{{SU}_{1}}}\text{max}\{\frac{\kappa_{2}}{\psi },\frac{\kappa_{1}}{1-\rho }\})\leq \varepsilon_{U_{1}}\\ \Rightarrow & \left\{ \begin{array}{cc} \rho \geq \frac{\kappa_{2}}{\left(1+\kappa_{2}\right)}-\frac{\kappa_{2}}{{\bar{\gamma }}_{s}\Omega_{{SU}_{1}}\left(1+\kappa_{2}\right)\text{ln}(1-\varepsilon_{U_{1}})}, & \rho \leq \varpi ,\\ \rho \leq 1+\frac{\kappa_{1}}{{\bar{\gamma }}_{s}\Omega_{{SU}_{1}}\text{ln}(1-\varepsilon_{U_{1}})}, & \rho >\varpi . \end{array}\right.\\ \Rightarrow & \underset{≜\vartheta_{h}}{\underset{︸}{1+\frac{\kappa_{1}/\left[{\bar{\gamma }}_{s}\Omega_{{SU}_{1}}\right]}{\text{ln}(1-\varepsilon_{U_{1}})}}}\geq \rho \geq \underset{≜\vartheta_{l}}{\underset{︸}{\frac{\kappa_{2}}{\left(1+\kappa_{2}\right)}[1-\frac{1/[{\bar{\gamma }}_{s}\Omega_{{SU}_{1}}]}{\text{ln}(1-\varepsilon_{U_{1}})}]}}. \end{array}$$ (46)

Besides, from (45d), (38), (42), (43), and (46), we can deduce the feasible region for *ρ* as

$$\begin{array}{c} \underset{≜\;\rho_{l}}{\underset{︸}{\text{max}\{\varphi ,\rho^{+},\varkappa ,\vartheta_{l}\}}}\leq \rho \leq \underset{≜\;\rho_{h}}{\underset{︸}{\text{min}\{1-\ell ,\theta ,\vartheta_{h}\}}}. \end{array}$$ (47)

On the other hand, we get that $R_{U_{1}}$ is a decreasing function of *ρ* as stated in **Remark** 6. Thus, *ρ* should be designed with a small value within the feasible region. By combining (46) and (47), the sub-optimal solution for the problem in (45) can be deduced as

$$\begin{array}{c} \rho^{⋆}=\{\begin{array}{cc} \rho_{l}, & \rho_{l}\leq \rho_{h},\\ \text{No}\;\text{solution}, & \rho_{l}>\rho_{h}. \end{array} \end{array}$$ (48)

## 4 Numerical results and discussions

In this section, we provide some numerical examples using the Monte-Carlo simulation with 10^4^ trials to validate the designed frameworks in the previous section. Specifically, we set the parameters throughout this section: *σ*^2^ = 1, $\Omega_{{SU}_{2}}=\Omega_{JE}=0.5$, and $\Omega_{{SU}_{1}}=\Omega_{SE}=1$.

### 4.1 Monitoring scenario

**Fig 2:**
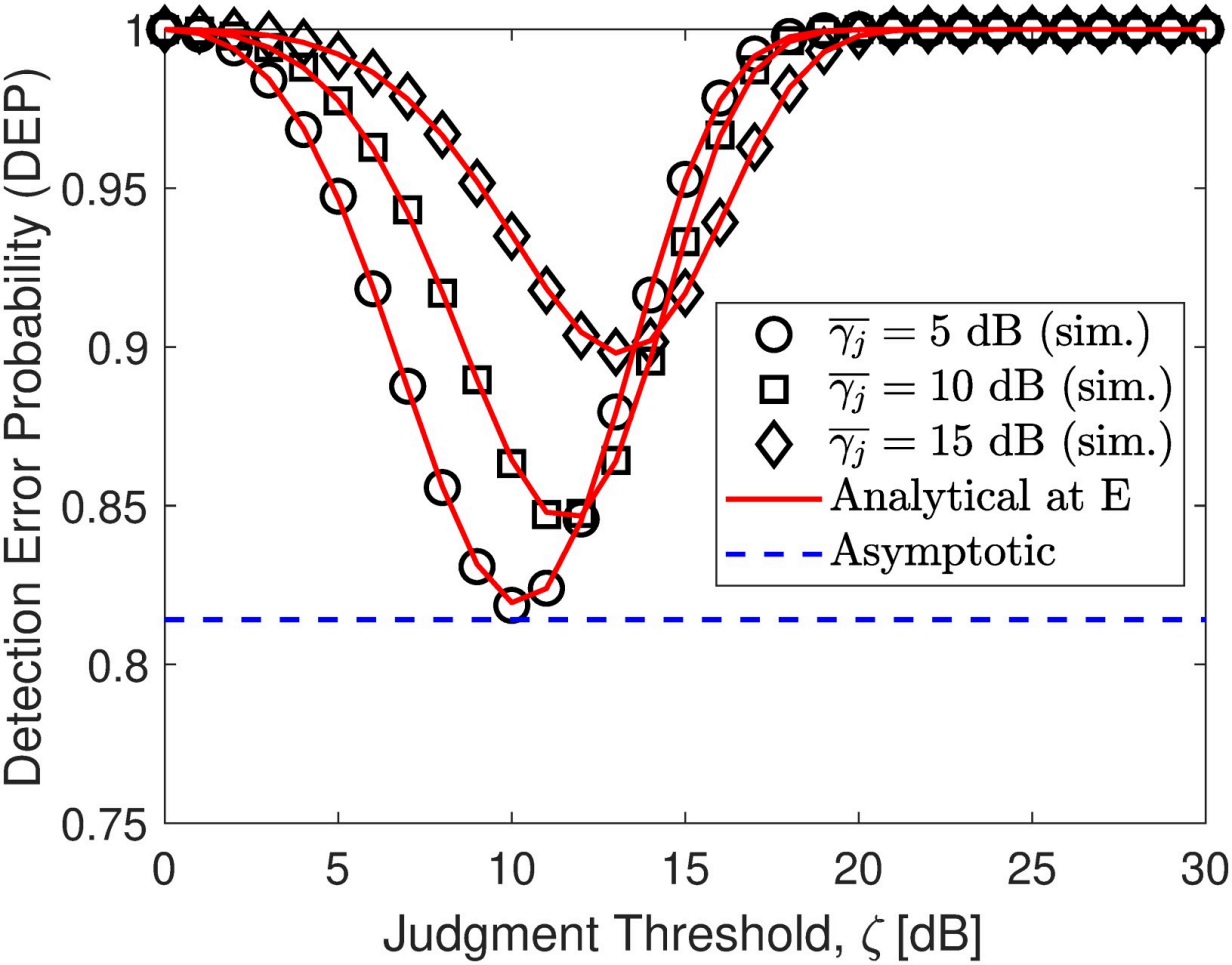
Impact of ${\bar{\gamma }}_{j}$ (jamming SNR) on DEP performance. Setups: a) *ρ* = 0.6 and ${\bar{\gamma }}_{s}=10$ dB, b) ${\bar{\gamma }}_{j}=5$ dB and ${\bar{\gamma }}_{s}=10$ dB, and c) *ρ* = 0.6 and ${\bar{\gamma }}_{j}=10$ dB.

**Fig 3:**
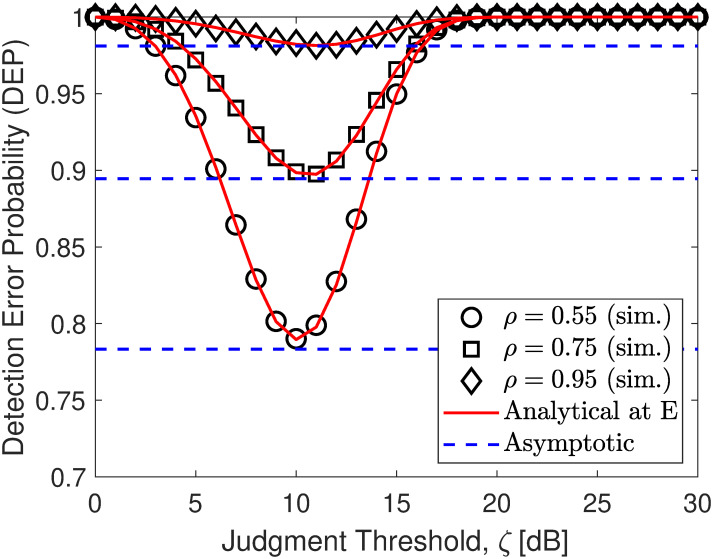
Impact of *ρ* (PA coefficient of $U_{2}$) on DEP performance. Setups: a) *ρ* = 0.6 and ${\bar{\gamma }}_{s}=10$ dB, b) ${\bar{\gamma }}_{j}=5$ dB and ${\bar{\gamma }}_{s}=10$ dB, and c) *ρ* = 0.6 and ${\bar{\gamma }}_{j}=10$ dB.

**Fig 4:**
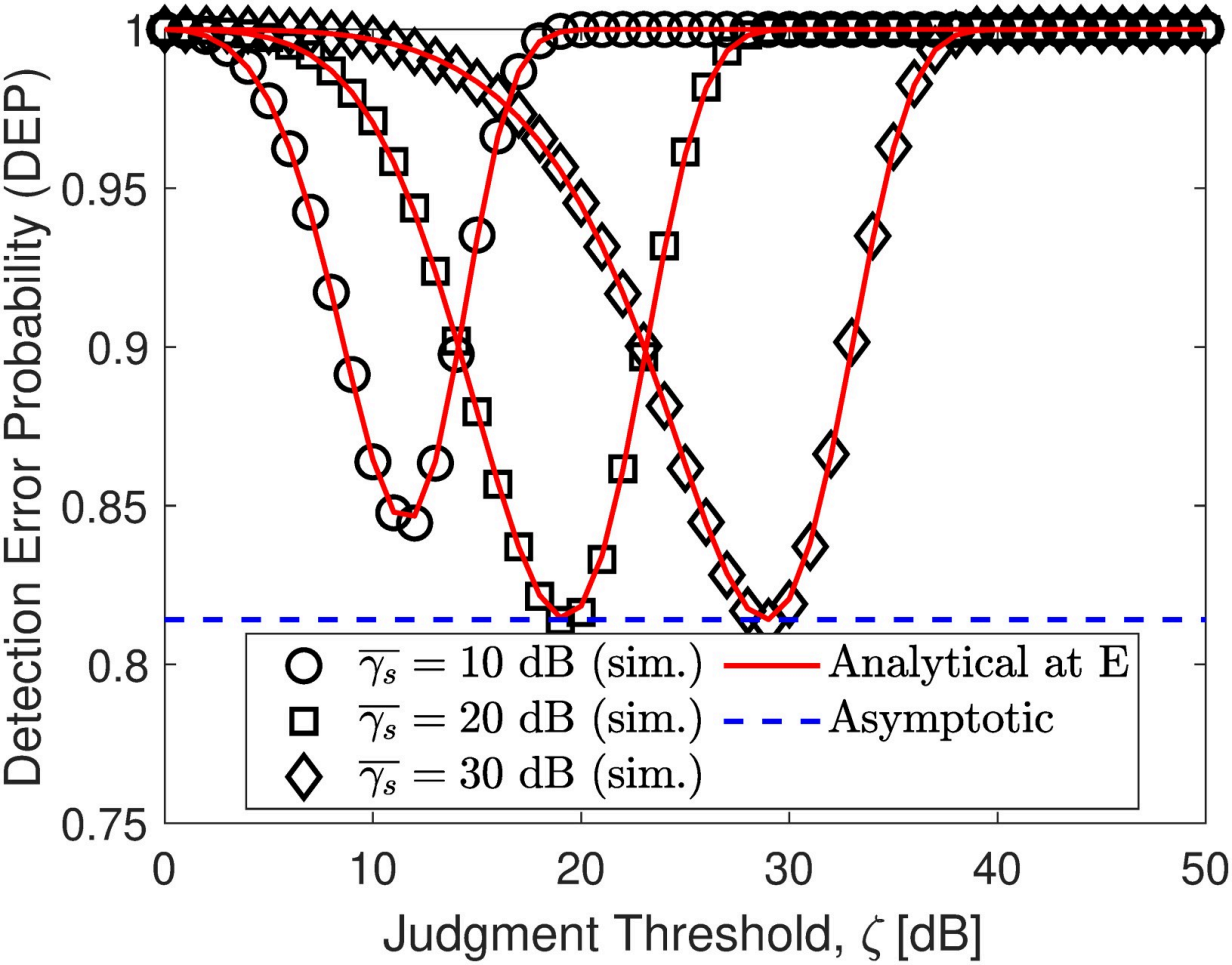
Impact of ${\bar{\gamma }}_{s}$ (transmit SNR) on DEP performance. Setups: a) *ρ* = 0.6 and ${\bar{\gamma }}_{s}=10$ dB, b) ${\bar{\gamma }}_{j}=5$ dB and ${\bar{\gamma }}_{s}=10$ dB, and c) *ρ* = 0.6 and ${\bar{\gamma }}_{j}=10$ dB.

In Figs 2–4, the DEP of monitoring covert transmission between S and $U_{1}$ for different adjustment activity of E to achieve the minimal DEP. As observed, both the simulation and analytical results in (10) are precisely consistent. Besides, we can find that the DEP has a downtrend with an increase in *ζ* and then becomes an uptrend in contrast. The reason is that when *ζ* increases, the probability of no transmission observed by E decreases but the probability of existent transmission of *x*_1_ observed by E increases and then the former becomes smaller than the latter, yielding an increase in DEP overall. Especially when they intersect, DEP reaches its minimum value, forming a downward concave shape. Fig 2 paints the DEP when ${\bar{\gamma }}_{j}=5,10,15$ dB. The results show that the amount of noise SNR strongly affects the DEP performance. When ${\bar{\gamma }}_{j}$ increases, E faces more error-prone in covert signal detection, yielding better covert protection of communication for the main system. Especially, we can see that the developed lower bound in (11) is almost lower than the minimal DEP, showing our correct mathematical development. Fig 3 shows the DEP with different values of power distribution coefficients *ρ*. It is observed that the higher the value of *ρ*, the larger the higher DEP performance. It can be concluded that the DEP increases with the increase of *ρ*. The covert performance is improved due to the application of efficient power distribution among NOMA signals that take advantage of user-superimposed messages to make friendly interference. Moreover, from Fig 4, we can further observe that when the transmit SNR ${\bar{\gamma }}_{s}$ increases, E must increase its threshold judgment to adapt to this change and from the developed asymptotic result, we can readily discover the minimum DEP that E can achieve without challenging.

**Fig 5:**
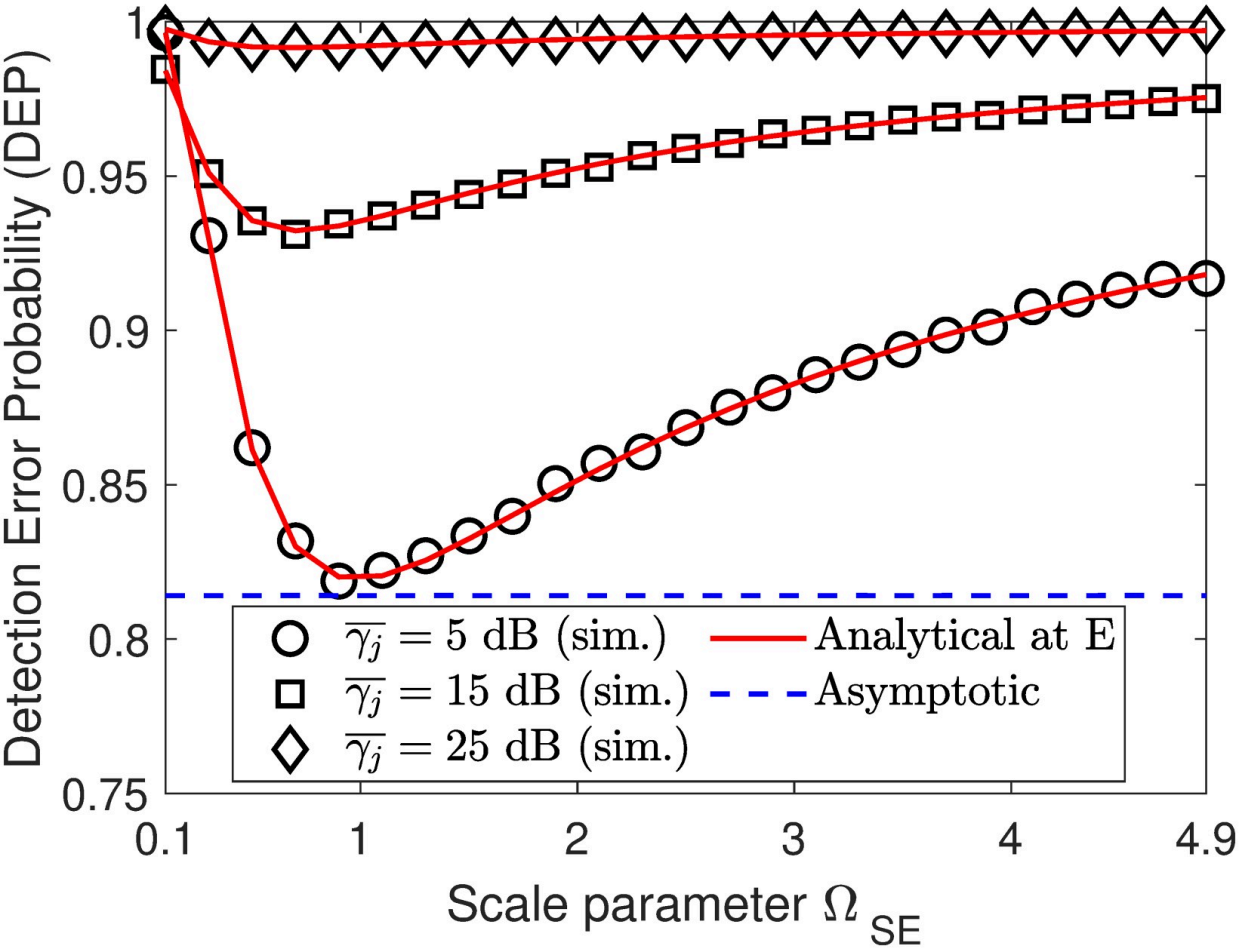
Impact of fading parameter $\Omega_{SE}$ on DEP performance. Setups: *ρ* = 0.6, ${\bar{\gamma }}_{s}=10$ dB, and *ζ* = 10 dB.

**Fig 6:**
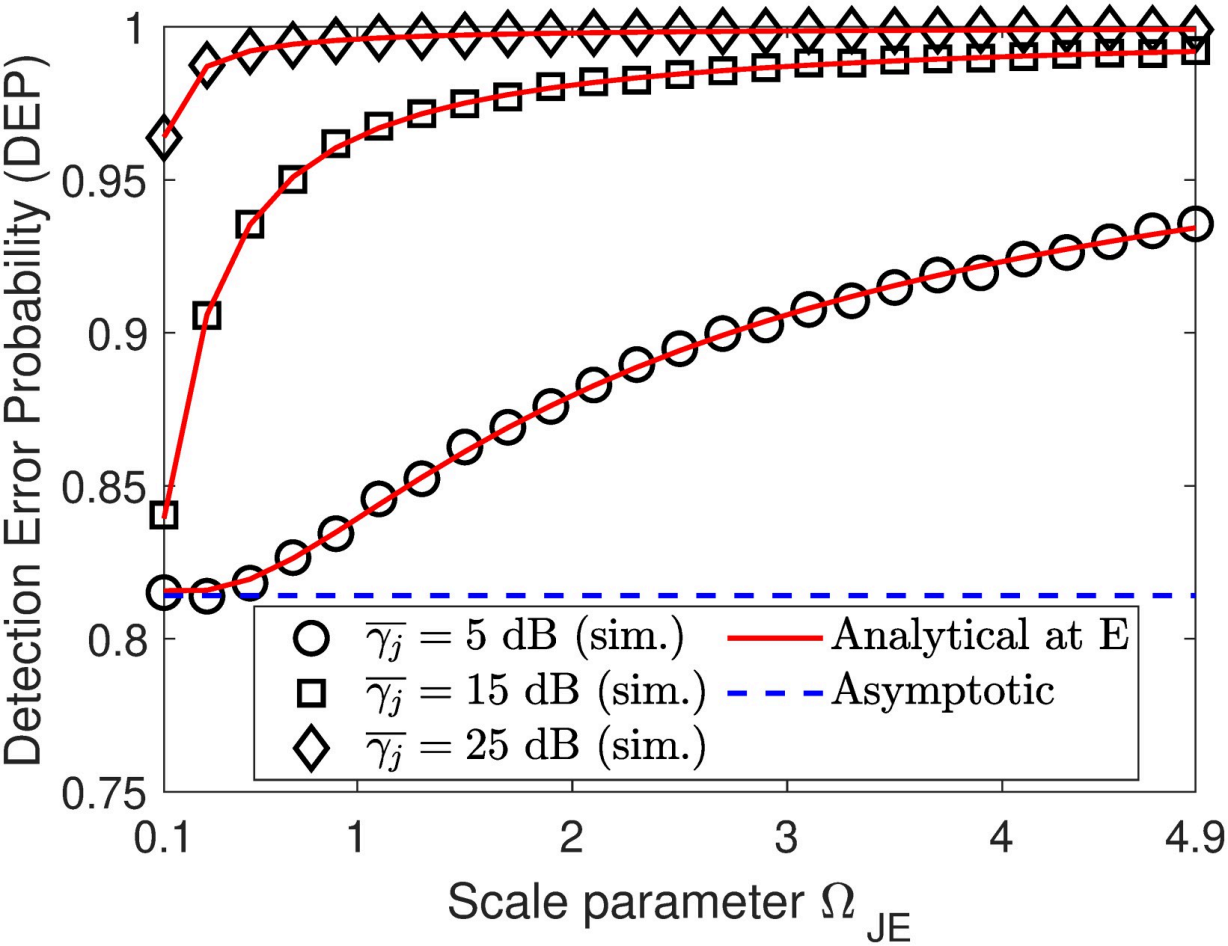
Impact of fading parameter $\Omega_{JE}$ on DEP performance. Setups: *ρ* = 0.6, ${\bar{\gamma }}_{s}=10$ dB, and *ζ* = 10 dB.

In Figs 5 and 6, we plot the DEP performance as a function of either $\Omega_{SE}$ or $\Omega_{JE}$. From Fig 5, we can observe that when $\Omega_{SE}$ increases, the DEP curves has downtrend with variations of $\Omega_{SE}$ from 0.1 to 1 and uptrend with variations of $\Omega_{SE}$ beyond 1. Meanwhile, the result in Fig 6 reveals that the DEP curves almost increase with an increase in fading parameter $\Omega_{JE}$. This is because the larger the value of $\Omega_{JE}$, the stronger the channel interference from the jammer node to the eavesdropper. Besides, we can also observe that increasing the jamming SNR ${\bar{\gamma }}_{j}$ plays an important role in increasing the DEP at the eavesdropper.

### 4.2 Eavesdropping scenario

**Fig 7:**
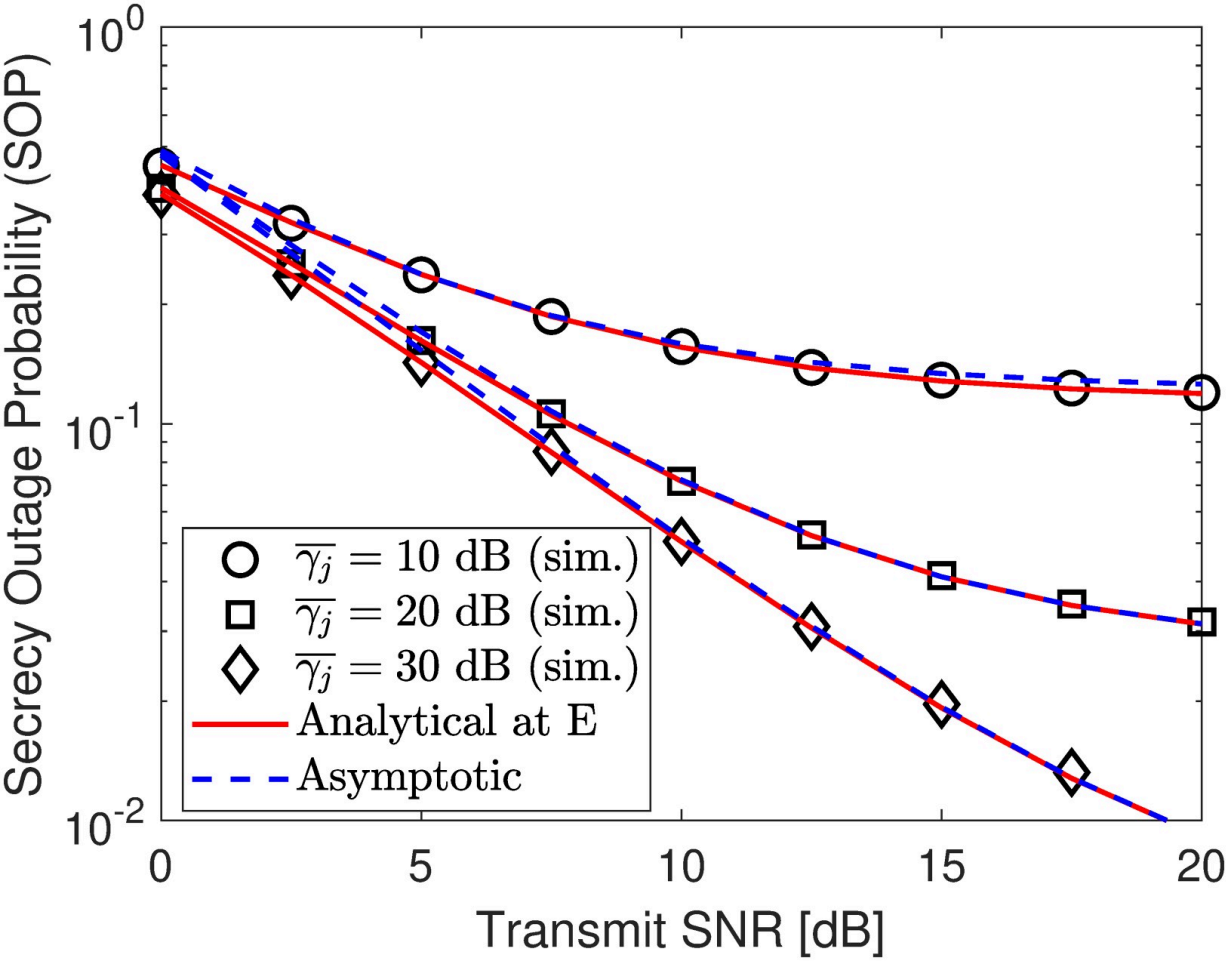
Impact of *ρ* (PA coefficient of $U_{2}$) on SOP performance under eavesdropping scenario. Setups: a) *R*_1_ = *r*_2_ = 0.25 bps/Hz and *ρ* = 0.6, b) *r*_2_ = 0.25 bps/Hz, ${\bar{\gamma }}_{j}=10$ dB, and *ρ* = 0.6, and c) *R*_1_ = *r*_2_ = 0.25 bps/Hz and ${\bar{\gamma }}_{j}=10$ dB.

**Fig 8:**
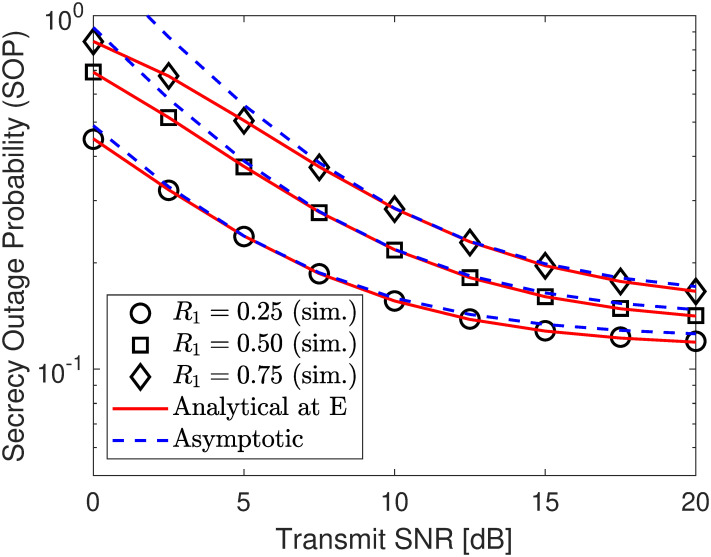
Impact of *R*_1_ (secure rate of $U_{1}$) on SOP performance under eavesdropping scenario. Setups: a) *R*_1_ = *r*_2_ = 0.25 bps/Hz and *ρ* = 0.6, b) *r*_2_ = 0.25 bps/Hz, ${\bar{\gamma }}_{j}=10$ dB, and *ρ* = 0.6, and c) *R*_1_ = *r*_2_ = 0.25 bps/Hz and ${\bar{\gamma }}_{j}=10$ dB.

**Fig 9:**
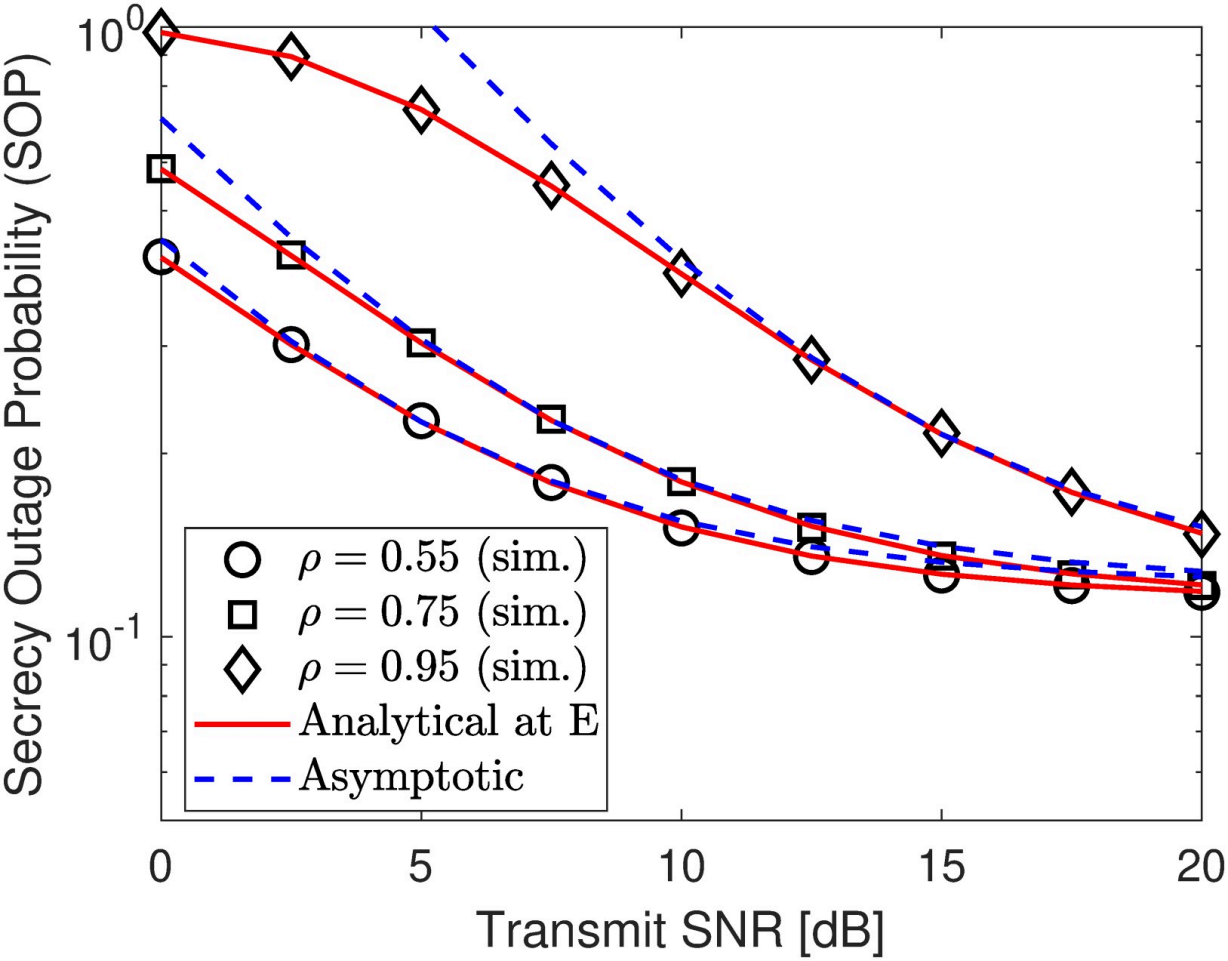
Impact of *ρ* (PA coefficient of $U_{2}$) on SOP performance under eavesdropping scenario. Setups: a) *R*_1_ = *r*_2_ = 0.25 bps/Hz and *ρ* = 0.6, b) *r*_2_ = 0.25 bps/Hz, ${\bar{\gamma }}_{j}=10$ dB, and *ρ* = 0.6, and c) *R*_1_ = *r*_2_ = 0.25 bps/Hz and ${\bar{\gamma }}_{j}=10$ dB.

Next, we depict the SOP under different jamming power levels in Fig 7, different target security rates in Fig 8, and other PA coefficient in Fig 9. According to the numerical results, no matter what SNR varies, the developed analytical results in (17) perfectly match with the simulation ones and approach the asymptotic plotted using (20) at high SNR region, verifying our developed analytical frameworks. According to the results in Fig 7, it can be seen that the security performance begins to improve and saturates the security floor with ${\bar{\gamma }}_{j}=10$ dB when ${\bar{\gamma }}_{s}$ exceeds 10 dBm. However, we can surpass this challenge by increasing jamming signal levels, i.e., ${\bar{\gamma }}_{j}$, to produce more interference to confuse E’s information extraction, strengthening the SOP significantly. Therefore, deploying a jammer has a splendid impact on security performance.

**Fig 10:**
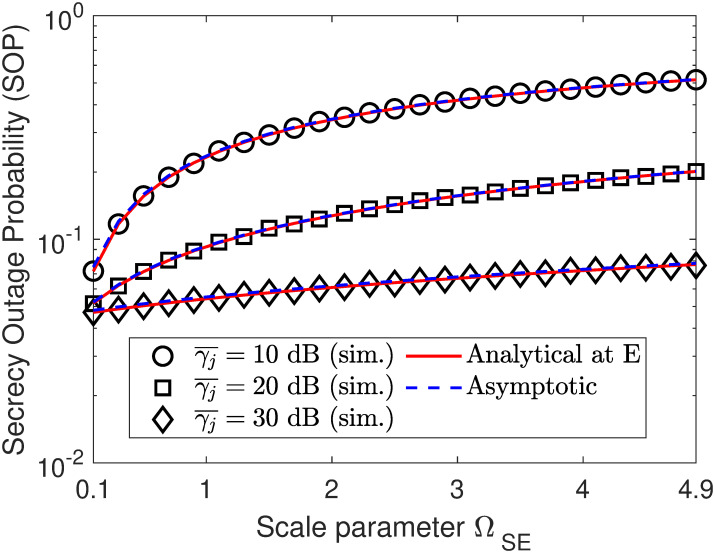
Impact of fading parameter $\Omega_{SE}$ on SOP performance. Setups: *R*_1_ = *r*_2_ = 0.25 bps/Hz, *ρ* = 0.6, and ${\bar{\gamma }}_{s}=10$ dB.

**Fig 11:**
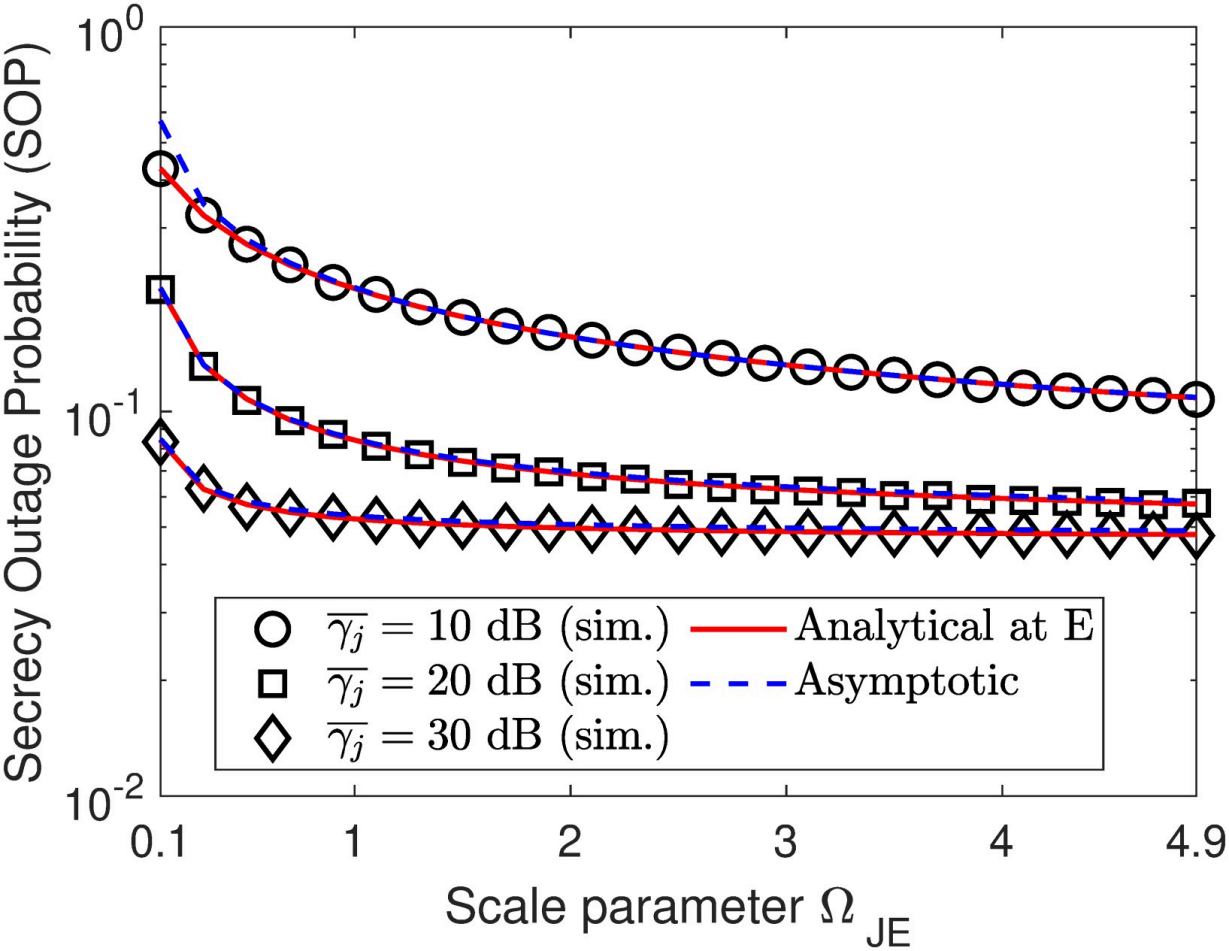
Impact of fading parameter $\Omega_{JE}$ on SOP performance. Setups: *R*_1_ = *r*_2_ = 0.25 bps/Hz, *ρ* = 0.6, and ${\bar{\gamma }}_{s}=10$ dB.

In Figs 10 and 11, we plot the SOP performance as a function of either $\Omega_{SE}$ or $\Omega_{JE}$. Specifically, we can observe that there is an opposite SOP trend between Figs 10 and 11, where the SOP in Fig 10 tends to increase with the increment of $\Omega_{SE}$, while that of Fig 11 tends to decrease with the increment of $\Omega_{JE}$. This is because when the eavesdropper is close to the source information (i.e., $\Omega_{SE}$ is increased), its channel gain becomes better and thus covert information of Bob can be decoded with higher probability. Meanwhile, increasing $\Omega_{JE}$ results in higher channel interference on the reception of the eavesdropper, thus degrading its decoding ability and improving the SOP accordingly. However, both also have the same trend is that increasing the jamming SNR ${\bar{\gamma }}_{j}$ helps improve the SOP performance, which is true with the expected role of the jamming node.

### 4.3 User performance

#### 4.3.1 Outage and rate analysis

**Fig 12:**
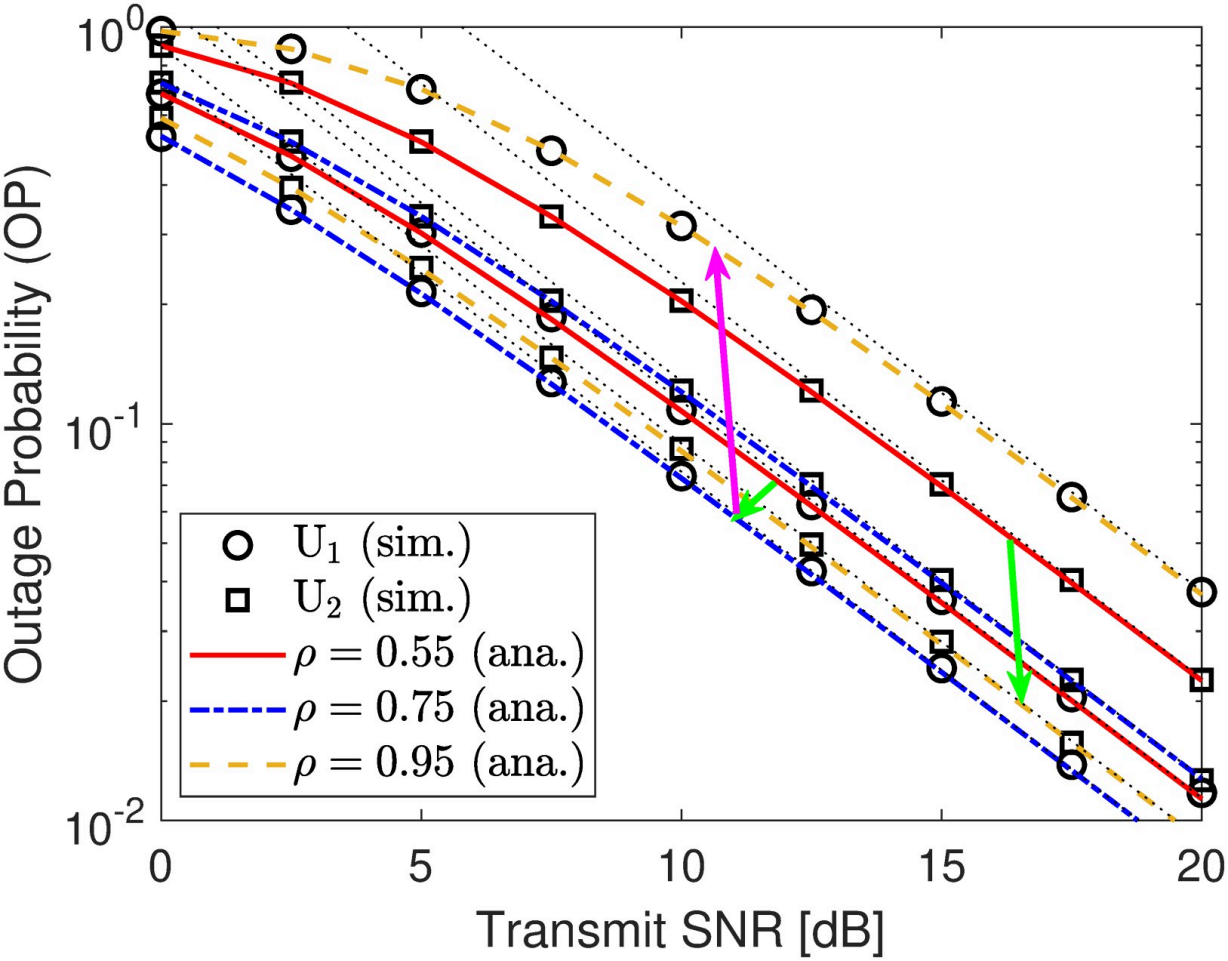
OP versus SNR ${\bar{\gamma }}_{s}$. Setups: a) *r*_1_ = 0.25 and *r*_2_ = 0.5.

**Fig 13:**
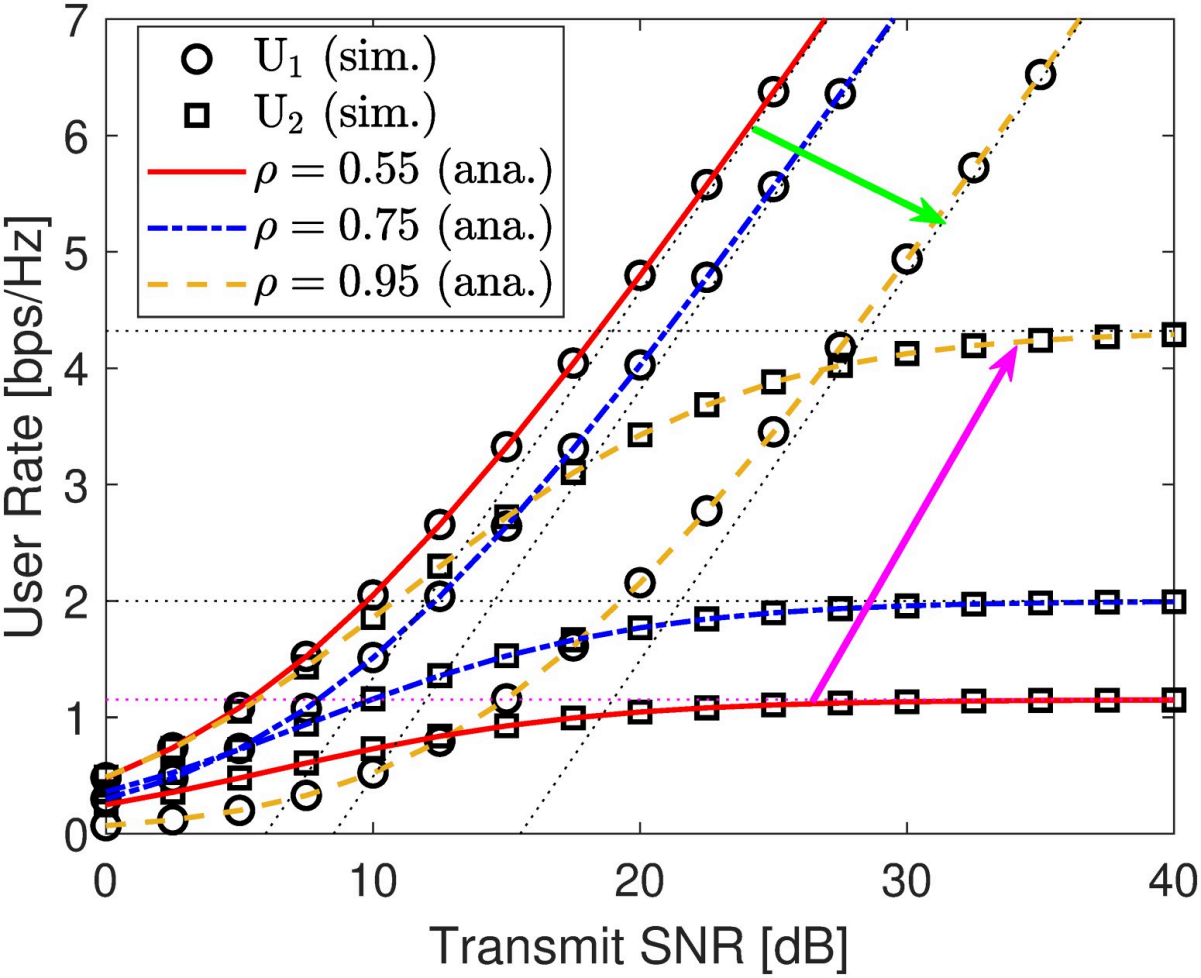
User rate versus SNR ${\bar{\gamma }}_{s}$. Setups: a) *r*_1_ = 0.25 and *r*_2_ = 0.5.

We investigate the relationship between the transmit SNR ${\bar{\gamma }}_{s}$ and the users’ OPs and rates. From Fig 12, it can be seen that no matter what the SNR changes, the OP curves improve considerably, and both simulation and analytical results are consistent with each other, showing the correctness of our derived expressions in (23) and (31). Besides, at high SNR, the asymptotic results (dot lines) can accurately predict the simulation ones, showing the correctness of (25) and (32). Moreover, we can further find from Fig 12 that the OP of $U_{2}$ almost improves with *ρ* = 0.55, 0.75, 0.95 when ${\bar{\gamma }}_{s}$ is constant. Conversely, the OP of $U_{1}$ has a downtrend with *ρ* = 0.55, 0.75 but an uptrend with *ρ* = 0.95. These observations are almost aligned with analyses in **Remarks** 4 and 7. Therefore, optimizing the power distribution coefficient *ρ* is a critical task. Next, from Fig 13, we can obtain the covert rate of $U_{1}$ corresponding to the linear curve of ${\bar{\gamma }}_{s}$ to its asymptote, where increasing ${\bar{\gamma }}_{s}$ has a strong effect in boosting the covert transmission rate. However, the rate of $U_{2}$ corresponds to the curve of ${\bar{\gamma }}_{s}$ to its asymptote, at which point increasing ${\bar{\gamma }}_{s}$ has almost no effect on the public transmission rate. However, when we increase the power distribution coefficient *ρ*, the public transmission rate improves considerably. Unfortunately, such configurations decline the quality of the covert transmission rate, yielding a trade-off in covert and public transmission rates between users.

#### 4.3.2 Covert outage minimization and rate maximization

**Fig 14:**
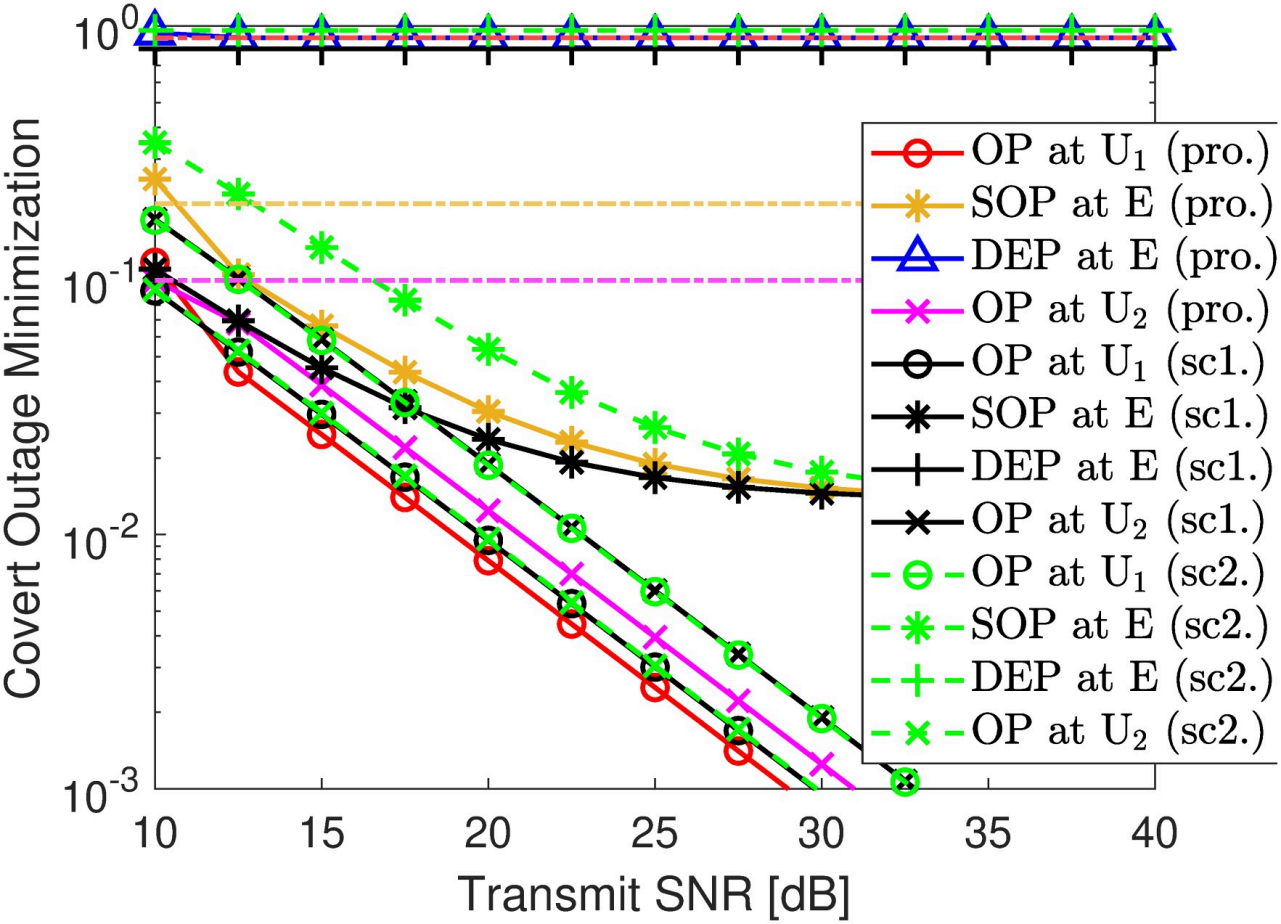
Cover outage optimization versus SNR ${\bar{\gamma }}_{s}$. Setups: a) *r*_1_ = 0.25 bps/Hz, *R*_1_ = *r*_2_ = 0.5 bps/Hz, ${\bar{\gamma }}_{j}=30$ dB, and *ℓ* = 10^−3^. b) *r*_1_ = 0.5 bps/Hz, *R*_1_ = 0.25 bps/Hz, ${\bar{\gamma }}_{j}=30$ dB, and *ℓ* = 10^−3^.

In Fig 14, the OP of transmitting a covert signal at $U_{1}$ is described as a function of the transmit ${\bar{\gamma }}_{s}$. By comparing two schemes of fixed PA scheme 1 (sc1.): *ρ* = 0.6 and scheme 2 (sc2.): *ρ* = 0.9. Under the constants for designing security $\epsilon_{E}=0.2$ (yellow dash-dot line), covert $\varrho_{U_{2}}=0.9$ (red dash-dot line), and reliability transmission $\varepsilon_{U_{2}}=0.1$ (pink dash-dot line), it can be seen that scheme 1 can satisfy the SOP at E and OP at $U_{2}$ with ${\bar{\gamma }}_{s}>12.5$ dB but violate the DEP requirement at E. Meanwhile, scheme 2 satisfies the SOP at E but requires ${\bar{\gamma }}_{s}>17.5$ dB to meet the OP at $U_{2}$ and ${\bar{\gamma }}_{s}>10$ dB to meet the SOP at E. Especially, compared to scheme 1, scheme 2 offers a better OP performance for $U_{2}$ but a lower OP performance for $U_{1}$. This trend is similar to that of Fig 14. This confirms the fact that distributing the coefficient *ρ* plays an important role in ensuring the system’s overall performance. With the proposed solution in (44), our approach does not only satisfy the DEP, SOP, and OP requirements but also improves the OP of $U_{1}$ considerably while achieving the OP of $U_{2}$ very close to that of scheme 2.

**Fig 15:**
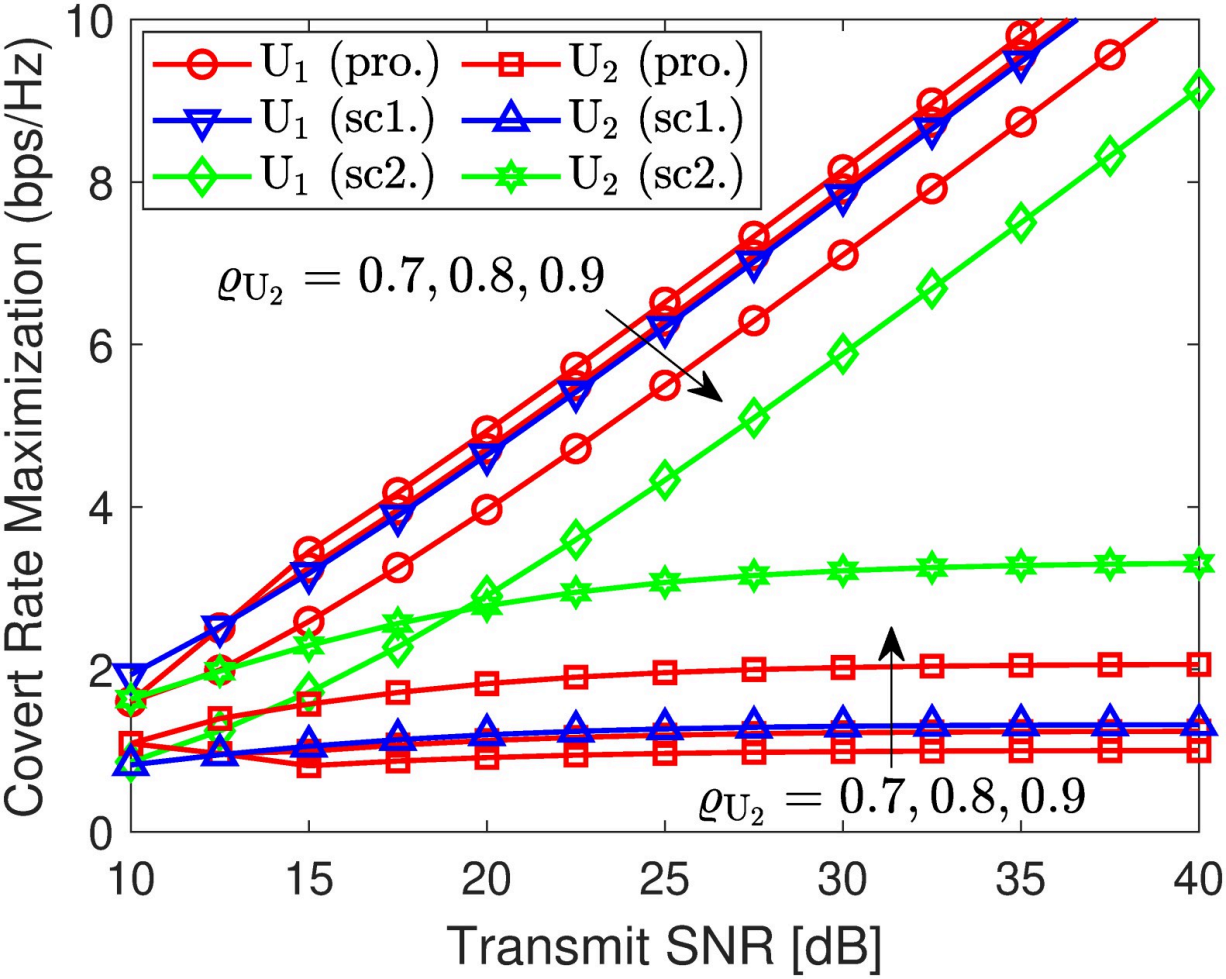
User rate optimization versus SNR ${\bar{\gamma }}_{s}$. Setups: a) *r*_1_ = 0.25 bps/Hz, *R*_1_ = *r*_2_ = 0.5 bps/Hz, ${\bar{\gamma }}_{j}=30$ dB, and *ℓ* = 10^−3^. b) *r*_1_ = 0.5 bps/Hz, *R*_1_ = 0.25 bps/Hz, ${\bar{\gamma }}_{j}=30$ dB, and *ℓ* = 10^−3^.

In Fig 15, the covert rate of $U_{1}$ is plotted as a function of the transmit ${\bar{\gamma }}_{s}$. As observed, exploiting scheme 2 provides a public rate for $U_{2}$ higher than Scheme 1 but results in a lower covert rage for $U_{1}$, and vice versa. However, schemes 1 and 2 do not consider the OP, SOP, and DEP requirements. Thus, if these constraints are accounted for covert communication and our proposed method in (48) is used, we can observe that when the target covert rate of $\varrho_{U_{2}}$ is small, the system has great change to allocate higher power level to enhance $U_{1}$’s covert rate. Specifically, when $\varrho_{U_{2}}=0.7$, the covert rate of $U_{1}$ can enhance up to 0.5 bps/Hz compared to Scheme 1 and 2 bps/Hz over Scheme 2. However, $\varrho_{U_{2}}=0.9$, the covert rate of $U_{1}$ is decreased since a lower PA level is required to meet the DEP demand as analyzed in **Remark** 1. Although the covert rate of $U_{2}$ is decreased, it remains higher than Scheme 2 and the public rate is indeed better than that of Scheme 1. These show that our proposed approach can efficiently capture the security risks and provide sustainable transmission with specific network conditions.

## 5 Conclusion

This work provided a comprehensive analysis of the NOMA system with users and external eavesdroppers in terms of outage transmission, secrecy outage performance and detection error. Under artificial noise generated by the jammer, closed-form for the OP, SOP, and DEP had been derived in terms of exact and asymptotic manner. Moreover, the covert and public rate formulas were also derived in exact and asymptotic. To enhance covert transmission, two optimization problems of covert outage minimization and covert rate maximization subject to the OP, SOP, and DEP requirements had been formulated and addressed, where we relied on the developed OP, SOP, and DEP to find the sub-optimal PA coefficient by closed-form expressions. Finally, we validated the developed mathematical and optimization frameworks by numerical results. Some key findings can be deduced from investigations of numerical result studies as follows:

1. Deploying the jamming node plays an important role in preventing potential external eavesdroppers. Specifically, increasing the jamming power not only causes more errors for the monitoring but also eavesdropping, leading to higher DEP and smaller SOP performances, respectively.
2. There exists a trade-off in allocating the transmit power between public and covert signals, where increasing the PA coefficient *ρ* almost improves the OP of $U_{2}$ but it yields a down-up trend on the OP of $U_{1}$. As for the ergodic rate aspect, increasing the PA coefficient *ρ* helps increase the capacity of $U_{2}$ but decreases that of $U_{1}$.
3. Given a fixed jamming power constraint, by exploring our proposed approach, the system can significantly improve the OP/cover rate of covert communication while ensuring the performance requirements of the OP of users, the SOP, and the DEP.

Since there is still much room that has not been covered yet, it is interesting to further investigate in the near future works, such as multi-antenna transmission, generalized channel models (e.g., Nakgami-m or Rician), untrusted near–trusted far user scenarios, multi eavesdroppers with colluding and non-colluding situations, imperfect channel estimation, and the issues of joint secure and covert energy efficiency. Besides, extending this study to backscatter communication [30] along with coverage enhancement with a reconfigurable surface can be regarded as a great potential direction for less-battery networks.

## Supporting information

S1 File The simulation code used in this work can be found at: MatlabCode. (ZIP)
